# Ultra‐Robust Conductive Hydrogels Enabled by a Gradient Bond‐Breaking Pseudo‐Drying Strategy

**DOI:** 10.1002/advs.202512144

**Published:** 2025-09-29

**Authors:** Dongchao Ji, Hongyang Han, Jiajun Li, Xiaoman Fei, Xiaolei Wang, Zhuochao Wang, Tao Song, Lei Yang, Zhibo Zhang, Wenxin Cao, Jiecai Han, Jiaqi Zhu

**Affiliations:** ^1^ National Key Laboratory of Science and Technology on Advanced Composites in Special Environments Harbin Institute of Technology Harbin 150001 P. R. China; ^2^ Zhengzhou Research Institute Harbin Institute of Technology Zhengzhou 450000 P. R. China; ^3^ School of Stomatology Harbin Medical University Harbin 150081 P. R. China

**Keywords:** anisotropic hydrogel, aramid nanofibers, cross‐scale networks, gradient bond‐breaking, polyvinyl alcohol, pseudo‐drying

## Abstract

Hydrogels, hydrophilic polymer networks mimicking biological tissues, hold great potential in biomedical applications such as electronic skin, tissue engineering, and biosensors. However, conventional hydrogels often struggle to concurrently achieve high strength, modulus, toughness, and fracture resistance, while dehydration and low‐temperature crystallization further limit their utility. Here, a biomimetic gradient bond‐breaking strategy is proposed to address these challenges. By constructing a hydrogel with covalently crosslinked hierarchical reinforcing phases—crystalline domains and self‐assembled aramid nanofibers (ANFs) networks—within the aqueous‐poor phase, preferential covalent bond rupture is enabled to enhance modulus, while hydrogen bond‐rich nanocrystalline domains and ANFs networks synergistically improve toughness. This mechanism yields fracture‐resistant properties akin to dry‐state materials, achieving a modulus of 12.4 MPa, toughness of 73.66 MJ m^−^^3^, and fracture toughness of 268.8 kJ m^−^^2^ at 70% water content—surpassing all reported PVA‐based hydrogels and even natural structural materials like tendon and spider silk. The mechanical properties are tunable via fabrication parameters, and the hydrogel exhibits broad‐temperature stability, high conductivity, and cytocompatibility. Leveraging these attributes, a low‐temperature‐operable strain sensor is developed for real‐time, accurate monitoring of human motion. This work advances the design of hydrogels with exceptional mechanical and functional properties for flexible electronics.

## Graphical abstract

A bioinspired PVA‐ANFs hydrogel, engineered via novel gradient bond‐breaking, achieves unprecedented mechanical performance: ultrahigh strength (≈18 MPa), modulus (≈12 MPa), toughness (≈74 MJ/m^3^), and record fracture energy (≈269 kJ m^−^^2^). It exhibits cryogenic flexibility (−70°C), high ionic conductivity (936.3 mS m^−1^), and biocompatibility. This breakthrough bridges synthetic‐natural material gaps, enabling applications in tissue engineering, biomedical devices, and flexible electronics.

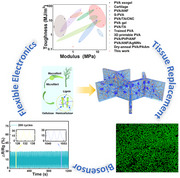

## 1 Introduction

Hydrogels are 3D network materials composed of hydrophilic polymers.^[^ ^1^, ^2^, ^3^, ^4^, ^5^, ^6^ ^]^ Their tissue‐like structure makes them promising for tissue engineering, drug delivery, and flexible electronics.^[^ ^7^, ^8^, ^9^, ^10^, ^11^, ^12^, ^13^, ^14^ ^]^ However, conventional hydrogel tensile curves are typically triangular in shape and often exhibit mechanical property fragility, limiting their use in load‐bearing or applications requiring high strength and toughness.^[^ ^15^, ^16^, ^17^ ^]^ Achieving simultaneous optimization of mechanical properties (strength, modulus, toughness, and fracture energy) and high‐water content remains a critical challenge in advanced hydrogel design. And with the development of hydrogel application scenarios, there are new requirements for its low‐temperature resistance.

Commonly used methods such as nanofillers, sacrificial bonds, and dual network structures can greatly enhance the toughness of hydrogels.^[^ ^1^, ^18^, ^19^, ^20^ ^]^ However, the aqueous‐phase lubrication effect and limited bonding varieties result in discontinuous stress transfer between reinforcing phases and the matrix.^[^ ^21^, ^22^ ^]^ This interfacial discontinuity leads to the persistent toughness‐modulus trade‐off that plagues most hydrogel systems.

Micro/nanostructure optimization offers a novel strengthening strategy.^[^ ^21^, ^23^, ^24^ ^]^ Hydrogels prepared by ice templating in combination with solvent substitution, thermal annealing or phase separation focus more on the microstructure of the gel than conventional methods. By utilizing abundant microcrystalline domains as the reinforcing phase, they can mimic the hierarchical microstructure of naturally tough materials (e.g., muscles, tendons, ligaments), avoiding stress concentrations and achieving synergistic performance enhancement.^[^ ^25^, ^26^, ^27^, ^28^, ^29^, ^30^, ^31^, ^32^ ^]^ Currently these methods show better performance in gel material systems such as PVA, gelatin, chitosan.^[^ ^30^ ^]^ However, inherent limitations persist. The bonding energy within the microcrystalline domains is low and molecular sliding between crystalline domains tends to limit the modulus of the gel (below 10 MPa). In contrast, mechanically trained hydrogels exhibit progressive alignment of polymer chains/nanofibers along the stress direction during cyclic stretching, effectively reducing chain entanglement disorder.^[^ ^33^, ^34^, ^35^ ^]^ This process generates a fiber‐reinforced composite‐like architecture that significantly suppresses molecular slippage during deformation. Consequently, the resulting mechanical behavior demonstrates ceramic‐like brittle fracture characteristics. This approach yields ultrahigh modulus (even greater than 100 MPa) but suffers from low water content, limited strain capacity, and poor toughness.

We note that wood achieves simultaneous high modulus and toughness through its oriented, cross‐scale architecture. The lignin plays a key role by effectively crosslinking cellulose and hemicellulose, significantly enhancing structural integrity.^[^ ^36^ ^]^ This interfacial bonding creates substantial steric hindrance during mechanical loading, leading to exceptional mechanical performance. This approach represents an effective solution to the longstanding trade‐off dilemma in mechanical properties. An analogous deformation mechanism is observed in solvent‐free polymer systems (e.g., polystyrene, polyurethane elastomers), characterized by an initial high‐modulus elastic deformation followed by pronounced yield behavior at large strains—precisely replicating the desired trapezoidal loading characteristics sought in hydrogels.^[^ ^37^, ^38^, ^39^ ^]^ This originates from immobilized rigid domains within the material, where the constrained interface between rigid and soft phases prevents molecular sliding, enabling high modulus. However, excessively strong interfaces may trigger brittle fracture upon initial deformation. Critically, these rigid domains maintain strength during stretching by avoiding catastrophic interface failure. Simultaneously, their progressive sliding (analogous to dislocation pile‐up in metals) induces strain hardening until ultimate fracture at high strain. All reported hydrogel materials exhibiting this mechanical behavior possess water contents below 40%.^[^ ^40^ ^]^ The fundamental constraint arises from an unresolved contradiction between structural optimization and high‐water content – excess water molecules act as lubricants during microstructural reorganization. This lubrication effect prevents the full realization of the unique structure's potential advantages.

In this work, we propose an innovative gradient bond‐breaking pseudo‐dry fabrication strategy to engineer high‐performance hydrogels. The design employs polyvinyl alcohol (PVA) as the matrix material, chosen for its exceptional cytocompatibility and tunable structural properties. Through directional freezing combined with a salting‐out process, we engineered an aqueous‐deficient phase structure containing densely packed crystalline microdomains. Concurrently, aramid nanofibers (ANFs)—capable of self‐assembling into nanoscale networks in protic solvents—are incorporated as a rigid secondary phase. Unlike conventional strategies focused solely on proportional modulation of microstructures, we emphasize the regulation of interactions among reinforcing phases. Covalent crosslinking was introduced between these phases to enhance structural integrity. Since the crosslinking occurs in the aqueous‐deficient regions, the hydrogel's water content remains unaffected. The covalent crosslinking endows the gel with high modulus and strength while preserving the molecular coiled conformation, thereby enhancing toughness. The resulting pseudo‐dry PVA‐ANFs (P‐PA) hydrogel exhibits remarkable properties: 70% water content, flexibility at −40 °C, ultrahigh strength (17.64 MPa), large strain (613.6%), exceptional toughness (73.66 MJ m^−^^3^), and outstanding fracture toughness (268.8 kJ m^−^^2^). Furthermore, the hydration of glycerol and inorganic salts effectively prevents hydrogel crystallization and hardening at extreme temperatures, while simultaneously conferring conductivity and cytocompatibility. This pseudo‐dry reinforcement strategy provides a feasible route to enhance the mechanical performance of high‐water‐content hydrogels, significantly broadening their application potential in demanding environments.

## 2 Results and Discussion

### 2.1 Fabrication of P‐PA Hydrogels Based on a “Pseudo‐Drying” Strategy

**Figure 1:**
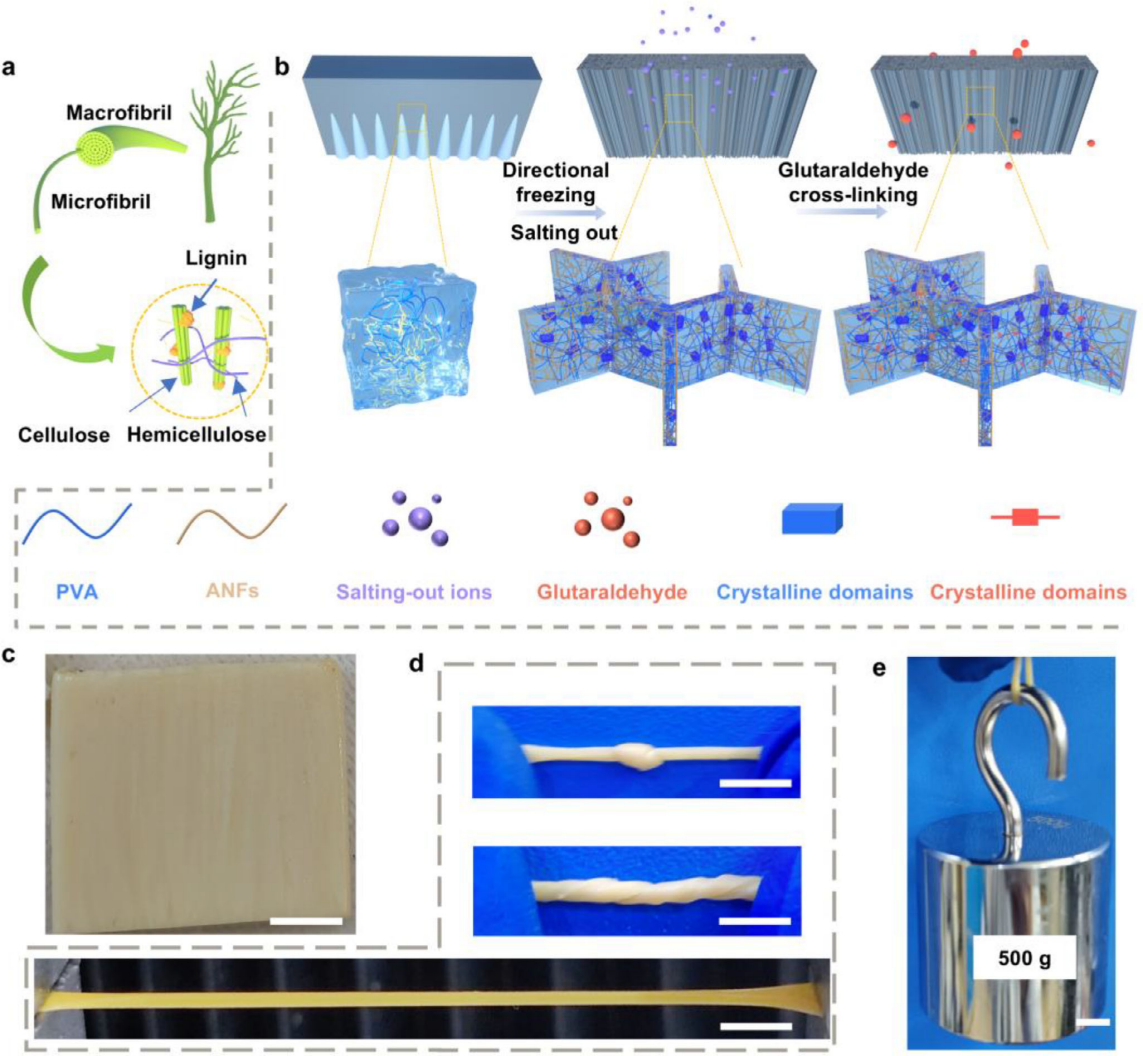
Inspired by wood fibers, a pseudo‐dry hydrogel was designed by directional freezing and Hoffmeister effect. a) Oriented multilevel crosslinked structure of natural wood fibers. b) Schematic preparation of the P‐PA hydrogel. c) Optical image of the prepared P‐PA hydrogel (scale bar = 1 cm). d) Photos of knotted, twisted and stretched hydrogels demonstrating flexibility (scale bar = 1 cm). e) Photo of a small piece of P‐PA hydrogel holding a 500 g weight. (scale bar = 1 cm).

Wood exhibits a unique combination of high modulus and toughness due to its hierarchically aligned, multiscale structure (**Figure** **1**a). Key to this performance is lignin, which acts as a natural adhesive, binding cellulose and hemicellulose into an integrated framework. The cohesive nature of lignin enhances structural integrity, while the large‐scale aligned architecture generates substantial steric hindrance under stress, leading to exceptional mechanical properties. Inspired by this mechanism, we developed a high‐strength, tough hydrogel by using sparse covalent bonds to anchor the reinforcing phase within the gel network (Figure 1b). First, we fabricated a micron‐scale aligned structure via ice‐templating. During directional freezing, the matrix phase (PVA) and reinforcing phase (ANFs) (Figure S1, Supporting Information) were expelled from growing ice crystals and confined to the intercolumnar gaps. Subsequent salting‐out induced the Hofmeister effect, driving PVA to form localized, water‐depleted crystalline domains, while protonated ANFs self‐assembled into a 3D network (Figure S2, Supporting Information). These two components were then covalently crosslinked via controlled glutaraldehyde treatment. In natural wood, dehydration enhances intermolecular interactions, drastically altering material properties. Our approach mimics this principle without sacrificing hydrogel hydration—hence termed the “pseudo‐dry” strategy—yielding a PVA‐ANFs hydrogel (P‐PA). The resulting P‐PA hydrogel exhibits a distinct aligned microstructure and an off‐white hue (Figure 1c). With good flexibility it can easily perform common behaviors such as knotting and twisting, while exhibiting a large deformation capacity (Figure 1d). Notably, it demonstrates exceptional load‐bearing capacity, supporting 500 g weights with a cross‐sectional area of just ≈1 mm^2^.

### 2.2 Structural Evolution of P‐PA

**Figure 2:**
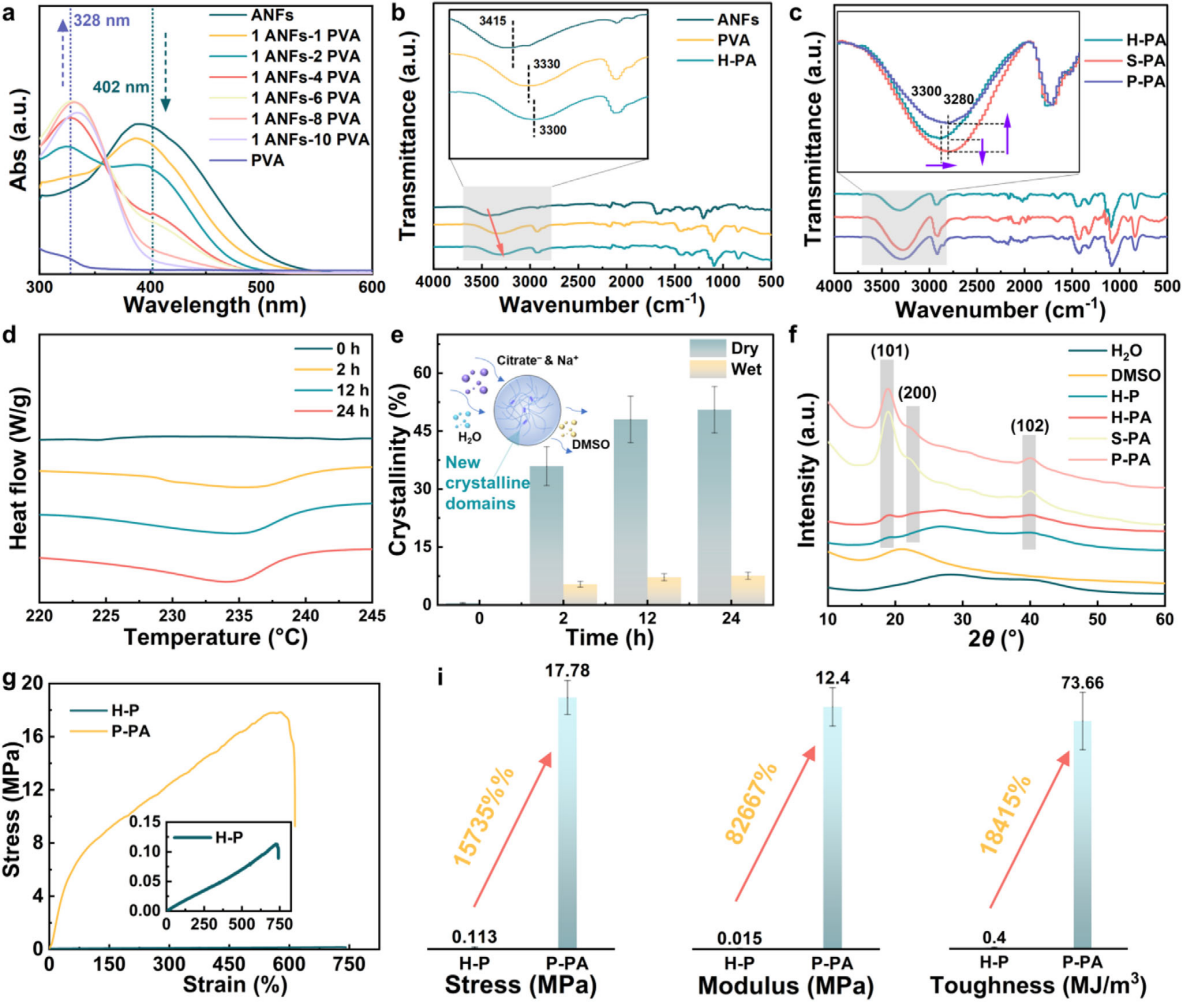
Structural evolution and toughening mechanism of P‐PA. a) UV–vis spectra and b) FT‐IR spectra for characterization of ANFs and PVA interactions. c) FT‐IR spectra of the salting‐out process and glutaraldehyde cross‐linking of P‐PA gels. d) DSC profiles and e) corresponding crystallinity of S‐PA gels treated with different times of salting out treatment. f) XRD patterns of the H‐P, H‐PA, S‐PA, and P‐PA. g) and h) A Comparison of mechanical properties of H‐P and P‐PA hydrogels. Data are expressed as mean ± standard deviation (*n* = 3 independent samples).

The interfacial interactions between the PVA matrix and aramid nanofiber (ANFs) reinforcements constitute the fundamental basis of the hydrogel's mechanical properties. UV–vis spectroscopy revealed that in ANFs/DMSO solutions, the ‐NH groups predominantly exist as N‐ ions. As shown in **Figure** **2**a, increasing PVA content leads to a gradual decrease in the N‐ ion absorption peak at 402 nm, accompanied by recovery of the NH peak at 328 nm. This spectral evolution demonstrates that PVA introduction disrupts the equilibrium of deprotonated ANFs solutions, thereby promoting ANFs self‐assembly. These observations were further corroborated by FT‐IR spectroscopy (Figure 2b). The characteristic peaks of ANFs (3415 cm^−1^) and PVA (3330 cm^−1^) underwent a significant redshift, merging into a single broad peak at 3300 cm^−1^ in the H‐PA hydrogel, indicating strong hydrogen bonding between the components. This PVA‐mediated surface modification of ANFs effectively screens the electrostatic repulsion in DMSO solution, facilitating ANFs self‐assembly into a 3D network. Subsequent salting‐out treatment with sodium citrate solution transformed H‐PA into S‐PA hydrogel. FT‐IR analysis revealed a further redshift of the hydroxyl peak from 3300 to 3280 cm^−1^ in S‐PA. After normalization against the CH_3_ peak, the enhanced intensity of the OH band confirmed increased hydrogen bonding density induced by the salting‐out effect. The pseudo‐dry P‐PA hydrogel, obtained through glutaraldehyde crosslinking, maintained the peak position but showed reduced intensity. This suggests consumption of hydroxyl groups via aldol condensation between glutaraldehyde and PVA's hydroxyl groups, while preserving the established hydrogen‐bonding network (Figure 2c). DSC analysis of S‐PA hydrogels at different salting‐out durations revealed that the crystallization growth rate began to slow after 24 h (Figure 2d,e), with crystallinity approaching 50%. XRD patterns indicated a minor crystalline phase in H‐P. Upon introducing ANFs, the (101) diffraction peak of PVA became more pronounced, suggesting that ANFs facilitated PVA crystallization. The crystallinity of PVA in S‐PA gels further increased significantly after salting‐out. In contrast, P‐PA gels crosslinked with glutaraldehyde showed no notable XRD pattern changes, implying that crosslinking primarily occurred among free hydroxyl groups. Collectively, these results demonstrate that P‐PA gels integrate a 3D nanofiber network of ANFs with pervasive PVA crystalline domains, while glutaraldehyde crosslinking intensified interfacial interactions among components. To elucidate the mechanism of gradient bond‐breaking, comparative XPS and FT‐IR analyses were performed on hydrogels subjected to 20% low‐strain cyclic loading (Figure S4, Supporting Information). The C═O bond—uninvolved in the rupture process—served as an internal reference, revealing a marked reduction in the relative ratios of C─C and C─O bonds post‐strain. This provides direct evidence for preferential scission of covalent bonds. Critically, FT‐IR spectra demonstrated minimal alterations in crystalline domains associated with hydroxyl absorption peaks, ruling out significant disruption of hydrogen‐bonded networks. These findings collectively indicate that covalent bonds undergo primary rupture during strain application. The presence of covalent bonds ensures enhanced overall resistance to stress, thereby strengthening the mechanical reinforcement effect in the low strain range. Consequently, P‐PA hydrogels exhibited exceptional mechanical properties, including a tensile strength of ∼18 MPa, elongation at break >600%, Young's modulus >12 MPa, and toughness of ≈74 MJ m^−^^3^ (Figure 2g). Figure 2h highlights the dramatic performance enhancement enabled by the pseudo‐dry strategy: P‐PA hydrogels achieved 15 735%, 82 667%, and 18 415% improvements in tensile strength, modulus, and toughness, respectively, compared to H‐P hydrogels.

### 2.3 Tuning the Mechanical Properties of P‐PA Hydrogels

**Figure 3:**
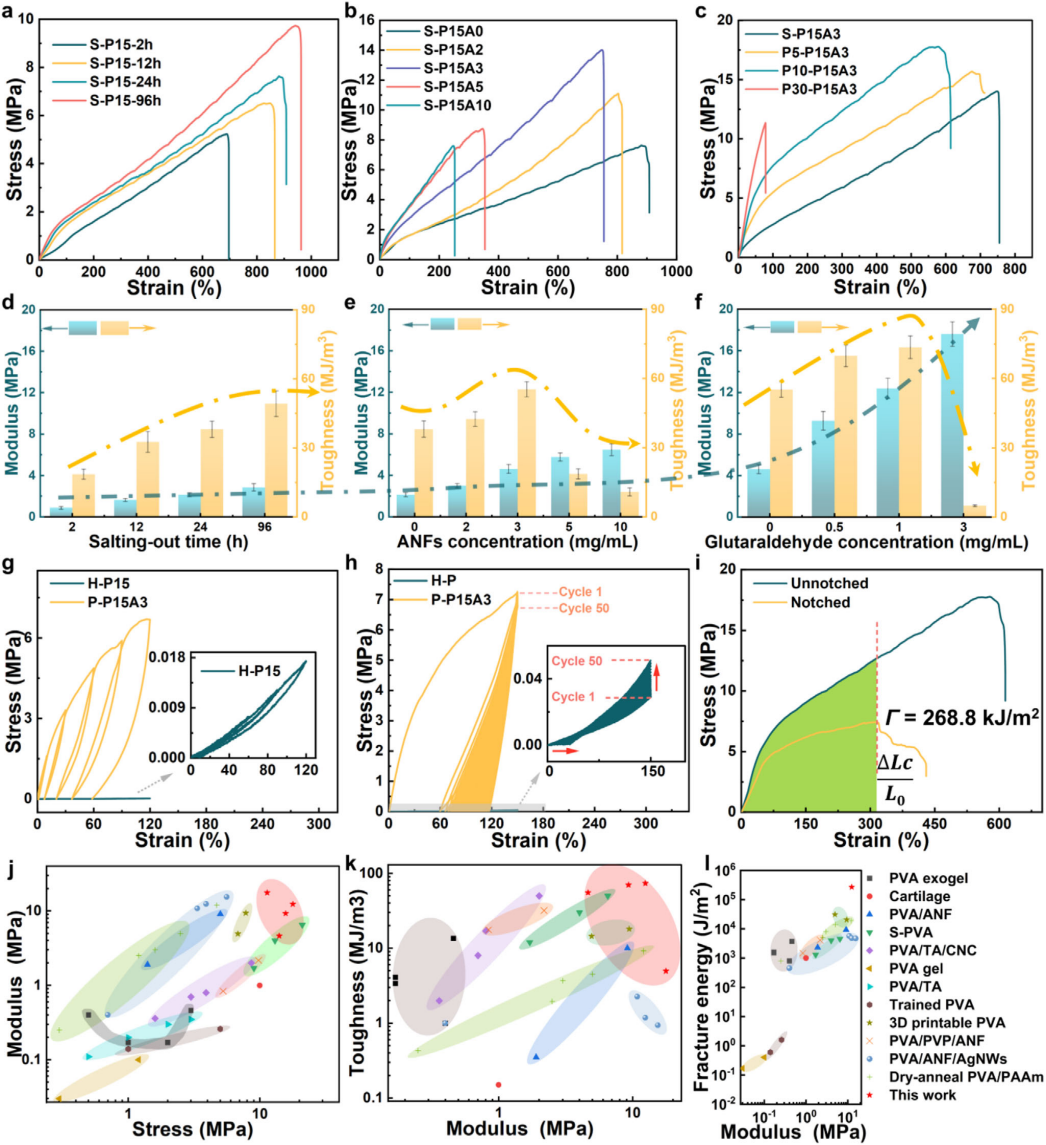
Quantitative characterization of the tensile mechanical properties of P‐PA hydrogels. Mechanical properties of a,d) S‐P hydrogels immersed in sodium citrate solution at varying salting‐out durations, b,e) S‐PA hydrogels with varying ANFs content, and c,f) P‐PA hydrogels with varying glutaraldehyde cross‐linking concentrations. g) Cyclic stress–strain responses of H‐P and P‐PA hydrogels with an incremental strain. h) Cyclic stress–strain responses of H‐P versus P‐PA hydrogels under 150% strain. i) Fracture energy of P‐PA hydrogel. j–l) Ashby charts of the mechanical properties of P‐PA hydrogel versus various other PVA‐based hydrogels and native soft tissues reported in the literature. (j) Comparison of tensile strength versus Young's modulus. (k) Comparison of Young's modulus versus toughness. (l) Comparison of Young's modulus versus fracture energy. PVA‐based hydrogels included the P‐PA hydrogel in current work, PVA exogel,^[^ ^27^ ^]^ PVA/ANFs,^[^ ^1^ ^]^ S‐PVA,^[^ ^31^ ^]^ PVA/Ta/CNC,^[^ ^44^ ^]^ PVA gel,^[^ ^45^ ^]^ PVA/TA,^[^ ^46^ ^]^ trained PVA,^[^ ^33^ ^]^ 3D printable PVA,^[^ ^47^ ^]^ PVA/PVP/ANFs,^[^ ^48^ ^]^ PVA/ANFs/AgNWs,^[^ ^49^ ^]^ and dry‐anneal PVA/PAAm.^[^ ^50^ ^]^ Native soft tissues were articular cartilage.^[^ ^43^ ^]^

The mechanical properties of P‐PA hydrogels are governed by PVA content, salting‐out duration, ANFs concentration, and crosslinking density. As demonstrated in **Figures** **3**a–f and S3 (Supporting Information), due to the short salting‐out time, the tensile curve of the hydrogel exhibits a triangular profile without a distinct yield point, indicating the absence of complex chain entanglements. The tensile strength and strain of S‐P hydrogels increased significantly from 0.48 MPa and 233% for H‐P7.5‐2h to 5.2 MPa and 700% for H‐P15‐2h. For H‐P20‐2h, the increased molecular content induced a transition in the tensile curve from triangular to trapezoidal shape. The modulus exhibited a substantial enhancement, rising from 0.6 MPa (H‐P7.5–2h) to 2.8 MPa. Concurrently, while the strength further increased, the deformation capability showed significant reduction. PVA content directly modulates molecular aggregation—higher concentrations promote stronger intermolecular interactions, thereby influencing the hydrogel's mechanical balance between strength and ductility. With increasing salting out time (from 2 to 96 h), the trapezoidal shapes of the stretching curves of the hydrogels are getting stronger. Prolonging the duration of salting out promotes the formation of additional crystalline domains, thereby enhancing intermolecular chain interactions. This results in a concurrent improvement in both strain capacity and tensile strength, along with increased modulus and toughness of the S‐P gels. For efficiency, a 24 h salting‐out duration was selected for subsequent gel preparations. Furthermore, self‐assembled 3D nanonetworks induced by protonation with ANFs significantly enhance the material's modulus. At optimal ANFs concentrations, the S‐PA hydrogel exhibits simultaneous gains in modulus and toughness, attributed to abundant hydrogen bonding between the ANFs and the PVA matrix. However, excessive ANFs loading leads to heterogeneous phase properties, ultimately degrading gel performance. Notably, the in situ‐formed 3D ANFs network functions as an efficient nanofiller, achieving optimal reinforcement at just 3 mg mL^−1^—demonstrating that high filler content is unnecessary for substantial mechanical enhancement. The directionally frozen gels showed significant anisotropy, which can be seen in Figure S5 (Supporting Information). However, even when stretched in the relatively weaker perpendicular direction, the S‐P15A3 hydrogel was as tough as previously reported tough hydrogels.^[^ ^41^, ^42^ ^]^ At this stage, the interactions between PVA crystalline domains and ANFs primarily rely on non‐covalent bonds. Inspired by the role of lignin in wood, moderate glutaraldehyde crosslinking was introduced to establish stronger connectivity between the reinforcing phase and the matrix. This method greatly improves the modulus and toughness of P‐PA gels with more pronounced trapezoidalization of the tensile curve. Notably, because crosslinking predominantly occurred in water‐deficient regions, the hydrogel maintained high water content while achieving mechanical properties comparable to those of dry‐state materials. The optimized PA hydrogel exhibited broadly tunable mechanical properties, with tensile strength ranging from 0.55 to 17.79 MPa, Young's modulus from 0.88 to 17.64 MPa, and toughness from 0.4 to 73.66 MJ m^−^^3^. As shown in Figure 3g–h, the P‐PA hydrogel demonstrated superior energy dissipation capability compared to the H‐P gel. Under cyclic loading, its modulus progressively decreased—a phenomenon attributed to the disruption of abundant crystalline domains during deformation, which effectively absorbed energy. This mechanism underpins the ultrahigh toughness of the P‐PA hydrogel. Remarkably, the P‐PA hydrogel demonstrated exceptional cyclic stability over 50 consecutive tensile cycles at fixed strain, alongside superior moisture retention. In contrast, the H‐P hydrogel exhibited significant performance degradation due to water loss during cycling. The anisotropic architecture of P‐PA, achieved via directional freezing, integrates high‐density crystalline domains with a 3D nanofibrillar ANFs network, endowing the material with crack‐insensitive behavior. This structural design yielded a fracture toughness of 268.8 kJ m^−^^2^ for P‐PA—a 256‐fold enhancement over the H‐P hydrogel (1.05 kJ m^−^^2^; Figures 3i; S6, Supporting Information). In addition to tensile properties, the P‐PA hydrogel exhibits excellent compressive performance, with its compressive strength increasing from 0.27 to 0.69 MPa at 65% strain. Moreover, it demonstrates stable compressive cyclic behavior under 40% strain (Figures S7 and S8, Supporting Information). Ashby‐type analysis (Figure 3j–l) further highlights the superiority of P‐PA, with its tensile strength, toughness, and Young's modulus exceeding those of most reported PVA‐based hydrogels and even surpassing native soft tissues (e.g., articular cartilage) (see Table S1, Supporting Information for detailed statistics).^[^ ^1^, ^27^, ^31^, ^33^, ^43^, ^44^, ^45^, ^46^, ^47^, ^48^, ^49^, ^50^ ^]^ These findings underscore that the pseudo‐dry strategy—targeted crosslinking of crystalline domains in water‐deficient regions via sparse covalent bonds—enables holistic reinforcement of the matrix, unlocking unprecedented mechanical properties.

### 2.4 Analysis of the Gradient Bond‐Breaking Mechanism of P‐PA Hydrogels

**Figure 4:**
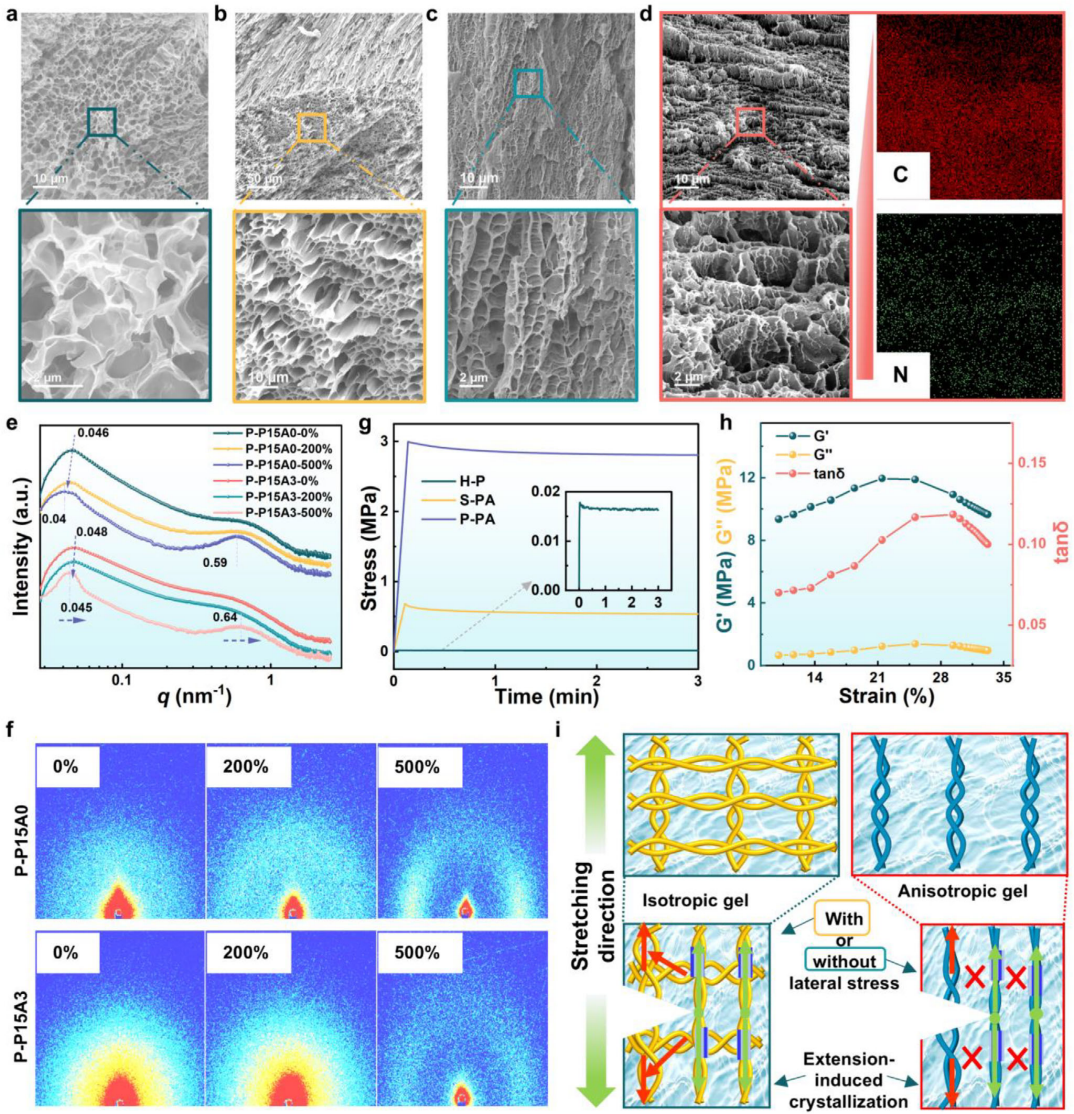
Analysis of the gradient bond‐breaking mechanism of P‐PA hydrogels. SEM images show the evolution in the microstructure of a) H‐P hydrogel, b) directional frozen H‐P hydrogel, c) S‐P hydrogel and d) S‐PA hydrogel. e) SASX profiles of the P‐P15A0 and P‐P15A3 at 0%, 200%, and 500% strain. f) SAXS patterns of P‐P15A0 and P‐P15A3 hydrogels during tensile loading. g) Creep behaviours of H‐P, S‐PA and P‐PA hydrogels. h) G′, G″, and tan δ of P‐PA gels from strain amplitude sweep (10%–33%) at a fixed angular frequency (10 rad s^−1^). i) Schematic depiction of crack‐tip stress field divergence between isotropic and anisotropic hydrogels.

In order to analyze the enhancement of mechanical properties by gradient bond‐breaking of biomimetic plant fibers, the studies were carried out in terms of cross‐section morphology, in situ small‐angle scattering and dynamic mechanics. **Figure** **4**a–d presents SEM images of the cross‐section morphologies for H‐P, D‐P, S‐P, and S‐PA hydrogels. The H‐P hydrogel exhibits an isotropic porous structure with smooth pore walls (Figure 4a, inset). In contrast, the D‐P hydrogel displays a highly aligned microstructure (Figure 4b), indicative of directional assembly. The S‐P hydrogel retains this oriented architecture, while salt precipitation induces the formation of PVA crystalline domains and in situ‐generated nanofibers along the pore walls (Figure 4c). This structural reorganization represents the first optimization step for enhancing the mechanical properties of the P‐PA hydrogel system. Upon incorporation of ANFs, energy dispersive spectroscopy (EDS) confirms the uniform distribution of nitrogen (N) within the pore walls (Figure 4d), verifying the successful self‐assembly of ANFs into a percolating 3D nano‐network. This nanofiber framework acts as a mechanical skeleton, marking the second reinforcement phase. Finally, glutaraldehyde cross‐linking selectively targets the water‐depleted regions of the hydrogel, integrating the two reinforcement phases (crystalline PVA and ANFs networks) into a cohesive hierarchical structure. This process yields a dry‐state‐like modulus while preserving high water content, as cross‐linking is confined to regions of poor water. Figure 4e reveals the presence of abundant crystalline domains in the P‐PA hydrogel. SAXS analysis shows that the scattering vector (q) decreases from 0.046 to 0.040 nm^−1^ for P‐P and from 0.048 to 0.045 nm^−1^ for P‐PA hydrogels during deformation. Concurrently, the radius of gyration (Rg) of the crystalline domains increases from 116 to 126 nm for P‐P and from 112 to 117 nm for P‐PA, demonstrating progressive dissociation of crystalline domains under strain – a key mechanism contributing to the exceptional toughness of P‐PA hydrogel. Notably, at 500% strain, new crystalline phases emerge at q = 0.59 nm^−1^ for P‐P and 0.65 nm^−1^ for P‐PA, providing direct evidence of strain‐induced crystallization and the associated hardening behavior. The higher q value observed for P‐PA suggests that ANFs promote the formation of smaller crystallites, reflecting strong interactions between ANFs and PVA chains. 2D SAXS patterns (Figure 4f) provide further insight into the structural evolution. Both P‐P and P‐PA hydrogels initially exhibit isotropic scattering halos characteristic of gradient structures. Upon stretching, the circular patterns progressively deform into elliptical shapes, indicating gradual alignment of polymer chains along the stretching direction. This anisotropic scattering evolution correlates with the macroscopic mechanical reinforcement observed during deformation. During the creep test (Figure 4g), the moderate covalent crosslinking in P‐PA gel ensures the structural integrity of the reinforcing phases. In the initial creep stage, the applied stress is not directly transferred to the sacrificial bonds due to this cohesive network. Consequently, the stress decay is mitigated, resulting in a higher modulus. In contrast, both S‐PA and H‐P gels lack covalent crosslinking, allowing the stress to directly act on the reinforcing phases. The rapid stress relaxation observed in these systems is attributed to the lubricating effect of water. However, S‐PA exhibits superior overall strength compared to H‐P due to the presence of large‐scale reinforcing phases (e.g., crystalline domains and ANFs networks), which induce pronounced steric hindrance. Dynamic strain sweep measurements demonstrate that both P‐P and P‐PA hydrogels exhibit markedly enhanced loss factors at initial strain compared to the H‐P hydrogel, suggesting continuous dissociation of crystalline domains under tensile deformation (Figures 4h; S9 and S10, Supporting Information). This elucidates the origin of the high toughness in P‐PA gels. Conventional isotropic hydrogels display crack sensitivity due to transverse stretching at the crack tip. In contrast, the directional freeze‐casting approach induces pronounced structural anisotropy, effectively suppressing transverse stress concentration and consequently endowing P‐PA hydrogels with exceptional fracture toughness.

### 2.5 Cytocompatibility Characterization of P‐PA Hydrogels

**Figure 5:**
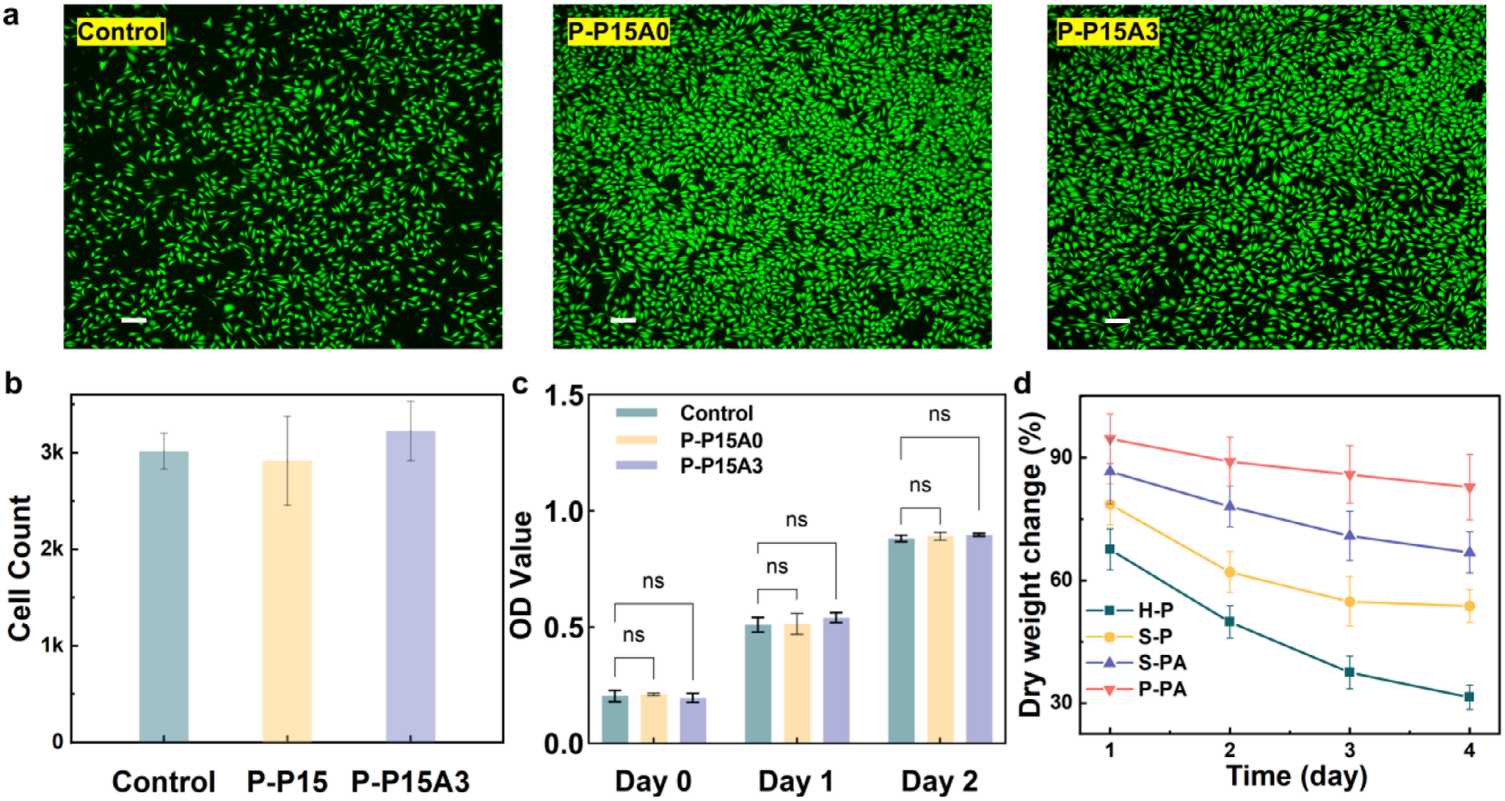
Cytocompatibility and degradation resistance of P‐PA hydrogels. a) live and dead cell staining assay and b) the corresponding number of cells after culturing for 48 h. Green and red represent the live and dead cells, respectively. Scale bar: 100 µm. c) Proliferation of L929 cells cultured on hydrogels for 24 and 48 h. d) Degradability of hydrogels in PBS solution.

The cytocompatibility of hydrogels is paramount for their potential biosensor applications. To evaluate this critical property, we systematically assessed the cytocompatibility of P‐PA hydrogels through live/dead cell staining assays and CCK‐8 proliferation tests using mouse fibroblast (L929) cells (**Figure** **5**a–c). Remarkably, the P‐PA hydrogel not only demonstrated excellent cell viability but also exhibited proliferative effects, with cell survival rates reaching 106.9% compared to control groups. The degradation resistance of P‐PA hydrogels, another crucial characteristic for biosensor and flexible electronics applications, was investigated through PBS immersion studies over four weeks (Figure 5d). The P‐PA hydrogel displayed outstanding stability against degradation, maintaining ≈ 82.8% of its original mass after the test period. This performance significantly surpassed that of untreated H‐P hydrogels, which retained only 31.4% mass. The initial rapid mass loss observed during the first two weeks can be attributed to the dissolution of uncross‐linked PVA components, after which the weight loss stabilized. Three key factors were identified to contribute to this enhanced stability: 1) the salting‐out effect, 2) incorporation of ANFs, and 3) glutaraldehyde crosslinking. Collectively, these results confirm the superior cytocompatibility and degradation resistance of P‐PA hydrogels, making them promising candidates for long‐term biomedical applications.

### 2.6 Flexible Sensing Application of the P‐PA Hydrogel in Cryogenic Environments

**Figure 6:**
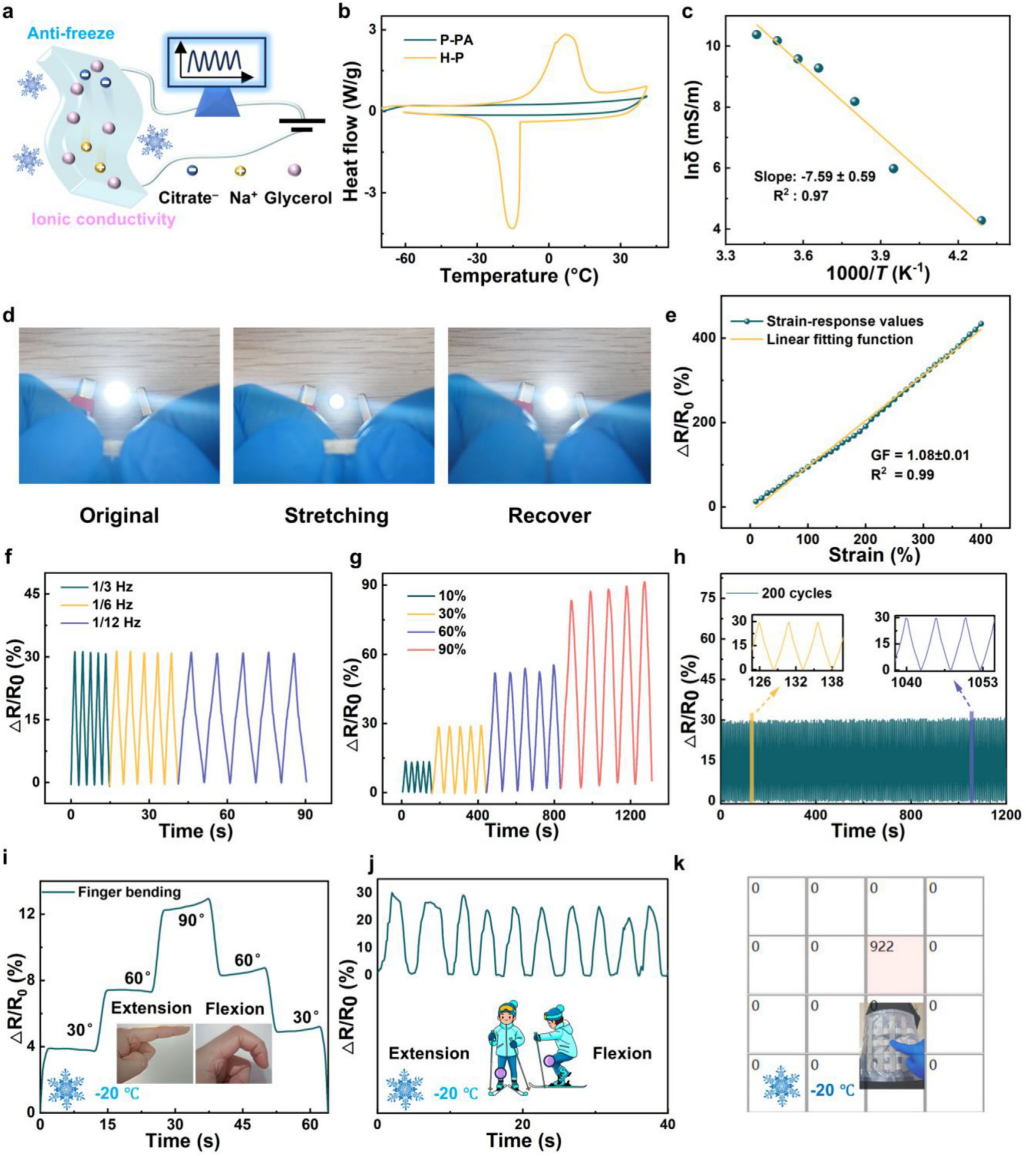
Flexible sensing performances of the P‐PA hydrogel. a) The advantages of P‐PA hydrogel. b) DSC analysis of H‐P and P‐PA hydrogel. c) Electrical properties of the P‐PA hydrogel with decreasing temperature. d) Complete circuit composed of an LED bulb and our ionic gel, showing the LED response at different strains. e) Sensitivity of P‐PA hydrogel sensor at −20°C. The P‐PA hydrogel sensor at −20 °C demonstrates: f) frequency‐dependent responses at 20% fixed strain, g) strain‐dependent responses under varying strains, and h) stable cyclic performance over 200 cycles at 20% strain. P‐PA hydrogel sensors for i) finger and j) knee flexion motion monitoring at −20 °C. k) Photograph of the E‐skin with a finger touching, and the corresponding pressure distribution. Unit: g.

Conventional hydrogels suffer from cryo‐crystallization, which significantly limits their applications in low‐temperature environments. The P‐PA hydrogel exhibits exceptional environmental tolerance and ionic conductivity, owing to the hydration effects of glycerol and salt ions. (Figure 5a). As shown in Figure 5b, the P‐PA hydrogel displays no water crystallization peak within the temperature range of −70–40°C, whereas the H‐P hydrogel demonstrates pronounced crystallinity. Specifically, the P‐PA hydrogel still has a deformation capacity of ≈ 300% at −40°C (Figure S11, Supporting Information). This indicates that the P‐PA hydrogel has good anti‐freezing properties. Moreover, the P‐PA hydrogel maintains excellent moisture retention, preserving 85% of its water content after 4 days of exposure to air before stabilizing. In contrast, the H‐P hydrogel retains only 11% under the same conditions (Figure S12, Supporting Information). The P‐PA hydrogel has good electrical conductivity because it contains a large number of conductive ions. According to Ohm's law, the conductivity of P‐PA hydrogel is 936.3 mS m^−1^. **Figure** **6**c shows the conductivity of P‐PA hydrogel at low temperature. Due to the low temperature stability of the P‐PA hydrogel, its conductivity at −40 °C is 72.24 mS cm^−1^ and the conductivity versus temperature follows the Arrhenius equation. Based on its good mechanical properties, environmental stability, and electrical conductivity, P‐PA hydrogel has the potential to be used as a sensor application. Upon stretching, the narrowing of ion migration channels and elongation of conduction pathways in the P‐PA hydrogel collectively increase the electrical resistance of the hydrogel. A direct visualization of strain responsiveness is achieved through an LED circuit, where the bulb's intensity diminishes at 200% strain due to increased resistance, and recovers upon stress release (Figure 6d). This establishes a well‐defined correlation between the relative resistance change (ΔR/R_0_) and applied strain, exhibiting a linear response with a gauge factor (GF) of 1.08 (Figure 6e). Notably, the sensor maintains highly stable and reproducible resistance signals at −20°C, demonstrating consistent ΔR/R_0_ variations and symmetrical response curves under various frequencies, different strains, and prolonged cyclic loading (Figure 6f–h). The outstanding strain‐responsive capability enables practical applications. Sensors mounted on fingers and knee joints reliably track joint motions at −20°C in outdoor conditions (Figure 6i,j). Furthermore, the hydrogel demonstrates potential as electronic skin. Inspired by neuronal arrays in biological skin, we fabricated an arrayed pressure sensor system. By assembling multiple micro‐pressure sensors into a multi‐pixel array configuration, spatial pressure mapping was achieved and validated through e‐skin prototype testing. Specifically, the P‐PA gel was pixelated into a 4 × 4 sensing array prototype with inter‐unit connections via conductive silver tapes. Each hydrogel cube (10 mm × 10 mm × 2 mm) functioned as an independent sensor unit/pixel. Finger pressing generated spatially resolved pressure distribution patterns across the array (Figure 6k). This shows the potential of the hydrogel as an electronic skin.

**Figure 7:**
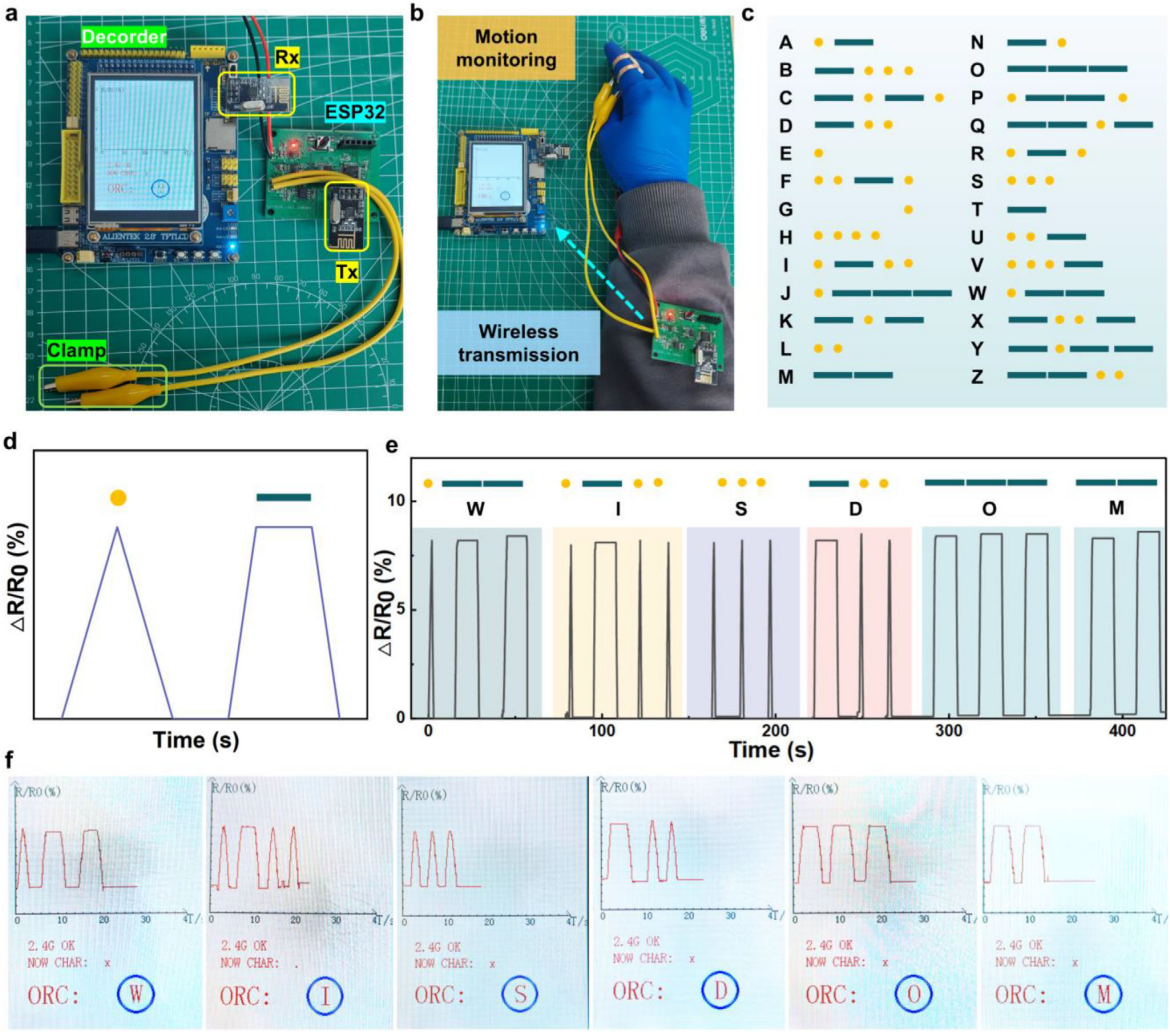
Demonstration of hydrogel‐based wireless communicator. a) A photograph showing the components of the self‐powered wireless transmission device. b) An example showing the working mechanism of the device by transmitting messages via finger motion. c) The definition of Morse code. d) The definition of the “dot” and “dash” signals by controlling the deformation time of the gel. e) The raw signals of different Morse codes by controlling the deformation time of the gel. f) Message displayed on the screen of the decoder by translating the raw signals into letters.

Building upon the conductive hydrogel's outstanding sensitivity and operational stability, we developed a self‐powered wireless transmission system (**Figure** **7**a). This integrated device couples with the hydrogel to transmit resistance variations during deformation to a decoder unit (Figure 7b), enabling real‐time conversion of electrical signals into legible alphabetic characters. The system represents Morse code through distinct combinations of “dot” and “dash” elements (Figure 7c). We established operational definitions where brief resistance fluctuations correspond to “dot” signals, while sustained resistance changes represent “dash” signals (Figure 7d). When mounted on various anatomical locations (particularly finger joints), the P‐PA hydrogel sensor reliably transduces limb movements into resistance variations. Through precise temporal control of deformation duration, the system achieves unambiguous discrimination between “dot” and “dash” signals, facilitating programmable information encoding via controlled signal sequences (Figure 7e). The decoder successfully demonstrated this functionality by converting electrical signals into visual text output, as evidenced by the accurate transmission of short messages (e.g., “WISDOM”) (Figure 7f). This indicates that the P‐PA hydrogel sensor has good signal transmission capability.

## 3 Conclusion

Inspired by plant fiber structures, we propose a pseudo‐dry strategy for fabricating hydrogels with high mechanical performance and cytocompatibility. The bioinspired hydrogel, prepared via a pseudo‐dry strategy involving gradient multi‐bond breakage, exhibits synergistic effects from anisotropic micron‐scale structures, in situ self‐assembled 3D nanonetworks, and selectively crosslinked crystalline domains. This enables high modulus, high energy dissipation, and crack insensitivity at high water content (70%), achieving a modulus of 12.4 MPa, toughness of 73.66 MJ m^−^^3^, and fracture toughness of 268.8 kJ m^−^^2^. The mechanical properties can be widely tuned by adjusting the fabrication process. Owing to the strong hydrogen bonding of glycerol and the hydration effect of inorganic salts, the hydrogel demonstrates excellent strain‐sensitive electrical properties and low‐temperature flexibility. Furthermore, it exhibits outstanding cytocompatibility and degradation resistance. Based on these remarkable features, the pseudo‐dry strategy‐derived hydrogel shows broad application potential in tissue replacement, electronic skin, and biosensors. More importantly, given the prevalence of Hofmeister effects or phase separation phenomena in various polymer‐solvent systems, we believe the pseudo‐dry strategy can be extended to diverse systems to advance practical applications.

## 4 Experimental Section

### Chemicals and Materials

Polyvinyl alcohol (PVA, MW = 89 000 g mol^−1^) was purchased from Sigma–Aldrich Co., Ltd. Sulfuric acid (98%) was purchased from Shanghai Lingfeng Chemical Reagent Co., Ltd. Kevlar‐49 was obtained from DuPont. Glutaraldehyde (50 wt.%) and dimethyl sulfoxide (DMSO, 99.7%) and potassium hydroxide (KOH, ≥90.0%) were purchased from Kelong Chemical Co. Cell Counting Kit‐8 (CCK‐8), calcein‐AM/PI double staining kit, and phosphate buffered solution (PBS) were purchased from Beyotime (China). (Shanghai). Dulbecco's modified Eagle's medium (DMEM) and fetal bovine serum (FBS) were purchased from HyClone Co., Ltd. L929 mouse fibroblast cells were purchased from Procell Co., Ltd. All chemicals were used directly without further purification.

### Preparation of Aramid Nanofibers

Aramid nanofibers (ANFs) were prepared according to previously established methods.^[^ ^51^ ^]^ In brief, 2 g of Kevlar‐49 fibers and 3 g KOH were added to 100 mL DMSO. This solution was magnetically stirred at room temperature for 7 days, and a dark red DMSO dispersion of ANFs (20.0 mg mL^−1^), marked as the mother liquor, was obtained.

### Preparation of Poly (vinyl alcohol) (PVA) Hydrogels

To prepare the PVA precursor solution, a measured amount of PVA powder was dissolved in dimethyl sulfoxide (DMSO) under vigorous stirring. The mixture was heated to 90 °C and stirred for 3 h until complete dissolution. The solution was then degassed via sonication for 1 h to ensure homogeneity and transparency. The PVA/DMSO solution was poured into a custom polytetrafluoroethylene (PTFE) mold with a copper block and subjected to directional freezing for 2 h using liquid nitrogen as the cooling medium. The ice‐templated PVA was subsequently immersed in a 40 wt.% sodium citrate aqueous solution for salting‐out, yielding the final directionally frozen PVA hydrogel (denoted as S‐Px, where x represents the PVA content). For comparison, PVA gels prepared without salting‐out were labeled D‐P, while hydrogels fabricated without directional freezing or salting‐out were designated H‐P.

### Preparation of Poly (vinyl alcohol) (PVA)‐ANFs Hydrogels

The fabrication process followed a similar procedure to that of pure PVA hydrogels, with the modification that PVA powder was dissolved in DMSO solutions containing varying concentrations of ANFs. The resulting composite hydrogels were designated as S‐PxAy, where x and y denote the respective weight fractions of PVA and ANFs. Control samples prepared without the salting‐out step were labeled H‐PA.

### Fabrication of Pseudo‐Dry PVA‐ANFs Hydrogels

The as‐prepared S‐PA hydrogels were immersed in glutaraldehyde (GA) solutions to induce covalent crosslinking. By modulating the GA concentration, the confinement strength of both ANFs nanonetworks and PVA crystalline microdomains within the system was precisely controlled, effectively mimicking the constrained microstructural states observed at varying degrees of dryness. Following crosslinking, the hydrogels were thoroughly dialyzed in deionized water containing glycerol and sodium citrate to remove residual GA and acidic byproducts. The final products were designated as Pk‐PxAy hydrogels, where k, x, and y represent the GA concentration, PVA content, and ANFs content, respectively.

### Microstructural Characterization

Fourier transform infrared spectra (FT‐IR) of the samples were collected with the FT‐IR spectrometer (PerkinElmer Frontier) in the frequency range of 4000–400 cm^−1^ with a total of 32 scans and resolution of 4 cm^−1^. The X‐ray diffraction (XRD) test was performed on x‐ray diffractometer (Ultima IV, Japan) instrument with an X‐ray of λ = 0.154 nm and the 2θ range of 5°–90°. The crystalline behavior and anti‐freezing properties of PVA hydrogels were measured on differential scanning calorimetry (DSC 214 Polyma, Germany) with a heating/cooling rate of 10 °C/min under nitrogen flow. Before DSC measurements, excess chemical crosslinks induced by glutaraldehyde were used to fix the amorphous PVA polymer chains in order to minimize formation of new crystalline domains during the drying process following.^[^ ^33^ ^]^ The small angle X‐ray scattering (SAXS) measurements were carried out at SAXS point 2.0 system (Anton Paar, Austria) with an X‐ray of λ = 0.154 nm. The sample‐to‐detector distance was 541 mm and the exposure time was set as 20 min. Transmission electron microscopy (TEM, Tecnai T20, FEI) was used to observe the morphology of the ANFs dispersions. The freeze‐dried hydrogels were brittlely exposed to cross sections with liquid nitrogen and sputtered with gold, followed by scanning electron microscope (SEM; HELIOS NanoLab 600i, FEI) test.

### Mechanical Testing

Hydrogels were shaped into dumbbell shapes for tensile testing at a tensile speed of 10 mm min^−1^. The specific dimension of each specimen was measured using calipers. The data were collected with an electronic universal testing machine (AGXplus, Shimadzu Corporate Management China Co., Ltd.).

The mechanical performance of hydrogels was quantitatively assessed through stress–strain analysis. Stress was calculated by dividing the applied load by the initial cross‐sectional area of the hydrogel specimen, while strain was determined as the displacement normalized to the initial gauge length. Two key parameters were derived: 1) the elastic modulus, obtained from the slope of the initial linear region (typically 0%–10% strain) in the stress–strain curve, representing the material's stiffness; and 2) toughness, calculated as the total area under the stress–strain curve, reflecting the energy required for fracture. Cyclic loading‐unloading tests were performed to evaluate the fatigue resistance and recoverability, with the hysteresis loop area quantifying the energy dissipation during each cycle. Fracture energy was determined following an established method, whereby tensile tests are applied on notched samples and compared with the results of unnotched samples for fracture energy calculations.^[^ ^52^ ^]^ Briefly, for a specimen with a cross‐sectional area *A*, span *L_0_*, *ΔL_c_*, is defined as the critical strain at which the notch becomes a running crack. *U*(*ΔLc*) is the work done on the unnotched specimen to achieve the *ΔLc* strain. The fracture energy can be given by Equation (1).

(1) $$\Gamma =U\left(\Delta Lc\right)/A$$

Dynamic mechanical analysis (DMA) of the P‐PA hydrogels were performed on DMA TA 850. The dynamic strain sweep test was performed at an angular frequency of 10 rad s^−1^.^[^ ^53^ ^]^

### Electrical Measurements

The electrical conductivities of the hydrogels (σ, S/m) were measured as Equation (2):^[^ ^53^ ^]^

(2) $$\sigma =\frac{L}{\mathrm{RA}}$$

where *L* (m) is the length of the hydrogel to be tested, *R* (Ω) is the electrical resistance, and *A* (m^2^) is the cross‐sectional area of the hydrogel.

**T**he resistance variation (Δ*R*/*R*_0_) and gauge factor (GF) of the hydrogels were calculated to evaluate the response performances of the conductive hydrogels using Equations (3) and (4), respectively:^[^ ^54^ ^]^

(3) $$\Delta R/R_{0}=\frac{R-R_{0}}{R_{0}}\times 100\%$$

(4) $$\mathrm{GF}=\left(\Delta R/R_{0}\right)/\varepsilon$$

where *R*_0_ and *R* represent the resistances without and with strain application, respectively, and ε is the strain of the hydrogels.

The specific testing process for the P‐PA gel sensor regarding frequency, strain, and cycling is as follows:

A reciprocating motion device and the sensor were sonnected in series to a circuit. In an environment at −20°C, the device is set to different motion frequencies (1/12, 1/6, and 1/3 Hz), different reciprocating amplitudes (10%, 30%, 60%, and 90%), and fixed at a strain of 30% for 200 reciprocating cycles. The device causes the sensor to deform along with it. The changes in the sensor's resistance can then be calculated based on the changes in voltage and current.

### Water Content

The water content (*C_W_*) of the hydrogels was calculated using Equation (5):

(5) $$C_{W}=\frac{W_{0}-W_{1}}{W_{0}}$$

Here, *W_0_* is the initial weight of the APP hydrogel, and *W_1_* is the weight of the hydrogel dried at 80°C for 2 h. All samples in the experiment had a water content of ≈ 70%.

### Cell Viability Analysis

The cytocompatibility of hydrogel extracts was evaluated using mouse fibroblast cells (NCTC clone 929, L929) via live/dead staining (Beyotime Biotechnology, China; Kit C2015M). A working solution was prepared by dissolving 9 µL propidium iodide (PI) and 9 µL calcein‐AM in 7.5 mL phosphate‐buffered saline (PBS). Following treatment with hydrogel extracts, cells were incubated with the staining solution for 30 min at 37 °C, washed three times with PBS, and immediately imaged using fluorescence microscopy. Calcein‐AM‐labeled live cells fluoresce green under 490 nm excitation and PI‐labeled dead cells fluoresce red under 535 nm excitation. Here, it is likely to be pointed out that for specific applications in implantable and wearable devices, direct co‐culture is more convincing than extraction metho.

### Cell Proliferation Assay

Cellular proliferation in hydrogel extracts was quantitatively evaluated at 24‐ and 48‐h intervals using a CCK‐8 colorimetric assay. The working solution was prepared by mixing CCK‐8 reagent with culture medium at a 1:10 (v/v) ratio. Subsequently, 110 µL of the working solution was added to each well of a 96‐well plate and incubated at 37 °C for 1 h. Absorbance was measured at 450 nm using a microplate reader (SpectraMax ABS Plus, Molecular Devices, USA). To ensure reproducibility, all experiments were performed in triplicate with three independent biological replicates. For cytocompatibility analysis, statistical significance between experimental and control groups at different time points was determined by one‐way ANOVA with post‐hoc Tukey's test (^*^ *p* < 0.05, ^**^ *p* < 0.01). Data represent mean ± standard deviation of three independent experiments. Here, it is likely to be pointed out that for specific applications in implantable and wearable devices, direct co‐culture is more convincing than extraction metho.

### Statistical Analysis

The data with an error bar were represented as mean ± standard deviation calculated on a minimum of three independent samples using the Origin software.

## Conflict of Interest

The authors declare no conflict of interest.

## Supporting information



Supporting Information

## Data Availability

The data that support the findings of this study are available in the supplementary material of this article.

## References

[1] L. Xu , X. Zhao , C. Xu , N. A. Kotov , Adv. Mater. 2018, 30, 1703343. 10.1002/adma.201703343 29134692

[2] X. Liu , J. Liu , S. Lin , X. Zhao , Mater. Today 2020, 36, 102.

[3] Y. Ding , H. Guo , M. Ouyang , G. Meng , F. Chen , T. Kuang , Adv. Funct. Mater. 2025, 35, 2421164.

[4] H. J. Kim , H. Kim , Y. H. Choi , E. S. Lee , Y. H. Kim , G.‐H. Lee , H. G. Chae , Y. Eom , ACS Nano 2025, 19, 8316. 39988896 10.1021/acsnano.4c18686

[5] C.‐C. Kim , H.‐H. Lee , K. H. Oh , J.‐Y. Sun , Science 2016, 353, 682. 27516597 10.1126/science.aaf8810

[6] X. P. Morelle , W. R. Illeperuma , K. Tian , R. Bai , Z. Suo , J. J. Vlassak , Adv. Mater. 2018, 30, 1801541. 10.1002/adma.201801541 29989671

[7] S. Liu , Y. Chen , J. Feng , J. Peng , Y. Zhou , Y. Zhao , Y. Zhao , Z. Lu , M. Sun , C. Wu , H. Hu , H. Rao , T. Zhou , G. Su , Chem. Eng. J. 2023, 466, 143087.

[8] C. Lim , Y. J. Hong , J. Jung , Y. Shin , S.‐H. Sunwoo , S. Baik , O. K. Park , S. H. Choi , T. Hyeon , J. H. Kim , S. Lee , D.‐H. Kim , Sci. Adv. 2021, 7, abd3716. 10.1126/sciadv.abd3716 PMC8104866 33962955

[9] J. L. Holloway , K. L. Spiller , A. M. Lowman , G. R. Palmese , Acta Biomater. 2011, 7, 2477. 21329769 10.1016/j.actbio.2011.02.016

[10] J. Zhang , L. Wan , Y. Gao , X. Fang , T. Lu , L. Pan , F. Xuan , Adv. Electron. Mater. 2019, 5, 1900285.

[11] Z. Wang , Z. Zhou , S. Wang , X. Yao , X. Han , W. Cao , J. Pu , Composites, Part B 2022, 239, 109954.

[12] M. Mehrali , A. Thakur , C. P. Pennisi , S. Talebian , A. Arpanaei , M. Nikkhah , A. Dolatshahi‐Pirouz , Adv. Mater. 2017, 29, 1603612. 10.1002/adma.201603612 27966826

[13] X. Wu , C. He , Y. Wu , X. Chen , Biomaterials 2016, 75, 148. 26497429 10.1016/j.biomaterials.2015.10.016

[14] H. Ceylan , I. C. Yasa , O. Yasa , A. F. Tabak , J. Giltinan , M. Sitti , ACS Nano 2019, 13, 3353. 30742410 10.1021/acsnano.8b09233 PMC6728090

[15] M. V. Chin‐Purcell , J. L. Lewis , J. Biomech. Eng. 1996, 118, 545. 8950659 10.1115/1.2796042

[16] M. Chen , J. Chen , W. Zhou , J. Xu , C.‐P. Wong , J. Mater. Chem. A 2019, 7, 26524.

[17] C. Shao , M. Wang , L. Meng , H. Chang , B. Wang , F. Xu , J. Yang , P. Wan , Chem. Mater. 2018, 30, 3110.

[18] C. Shao , L. Meng , M. Wang , C. Cui , B. Wang , C.‐R. Han , F. Xu , J. Yang , ACS Appl. Mater. Interfaces. 2019, 11, 5885. 30652853 10.1021/acsami.8b21588

[19] R. Zhu , Z. Zheng , D. Zhu , X. Wang , J. Colloid Interface Sci. 2025, 677, 687. 39116566 10.1016/j.jcis.2024.08.008

[20] J. P. Gong , Y. Katsuyama , T. Kurokawa , Y. Osada , Adv. Mater. 2003, 15, 1155.

[21] Y. Hou , M. Jin , Y. Liu , N. Jiang , L. Zhang , S. Zhu , Chem. Eng. J. 2023, 460, 141731.

[22] T. Shoaib , R. M. Espinosa‐Marzal , Colloids Interfaces 2020, 4, 54.

[23] J. Bae , Y. Li , J. Zhang , X. Zhou , F. Zhao , Y. Shi , J. B. Goodenough , G. Yu , Angew. Chem., Int. Ed. 2018, 57, 2096. 10.1002/anie.201710841 29314472

[24] Z. Zhao , R. Fang , Q. Rong , M. Liu , Adv. Mater. 2017, 29, 1703045. 10.1002/adma.201703045 29059482

[25] X. Liang , G. Chen , S. Lin , J. Zhang , L. Wang , P. Zhang , Z. Wang , Z. Wang , Y. Lan , Q. Ge , J. Liu , Adv. Mater. 2021, 33, 2102011. 10.1002/adma.202102011 34110665

[26] S. Wu , M. Hua , Y. Alsaid , Y. Du , Y. Ma , Y. Zhao , C.‐Y. Lo , C. Wang , D. Wu , B. Yao , J. Strzalka , H. Zhou , X. Zhu , X. He , Adv. Mater. 2021, 33, 2007829. 10.1002/adma.202007829 33554414

[27] L. Xu , S. Gao , Q. Guo , C. Wang , Y. Qiao , D. Qiu , Adv. Mater. 2020, 32, 2004579. 10.1002/adma.202004579 33169449

[28] H. Bodugoz‐Senturk , J. Choi , E. Oral , J. H. Kung , C. E. Macias , G. Braithwaite , O. K. Muratoglu , Biomaterials 2008, 29, 141. 17950839 10.1016/j.biomaterials.2007.09.015

[29] M. Hua , S. Wu , Y. Ma , Y. Zhao , Z. Chen , I. Frenkel , J. Strzalka , H. Zhou , X. Zhu , X. He , Nature 2021, 590, 594. 33627812 10.1038/s41586-021-03212-z

[30] S. Wu , Z. Liu , C. Gong , W. Li , S. Xu , R. Wen , W. Feng , Z. Qiu , Y. Yan , Nat. Commun. 2024, 15, 4441. 38789409 10.1038/s41467-024-48745-9 PMC11126733

[31] D. Liu , Y. Cao , P. Jiang , Y. Wang , Y. Lu , Z. Ji , X. Wang , W. Liu , Small 2023, 19, 2206819. 10.1002/smll.202206819 36592418

[32] B. Bao , Q. Zeng , K. Li , J. Wen , Y. Zhang , Y. Zheng , R. Zhou , C. Shi , T. Chen , C. Xiao , B. Chen , T. Wang , K. Yu , Y. Sun , Q. Lin , Y. He , S. Tu , L. Zhu , Nat. Mater. 2023, 22, 1253. 37604908 10.1038/s41563-023-01648-4

[33] S. Lin , J. Liu , X. Liu , X. Zhao , Proc. Natl. Acad. Sci. USA 2019, 116, 10244. 31068458 10.1073/pnas.1903019116 PMC6535018

[34] M. Sun , H. Li , Y. Hou , N. Huang , X. Xia , H. Zhu , Q. Xu , Y. Lin , L. Xu , Sci. Adv. 2023, 9, ade6973. 10.1126/sciadv.ade6973 PMC9937573 36800416

[35] C. Xu , A. Xie , H. Hu , Z. Wang , Y. Feng , D. Wang , W. Liu , Nat. Commun. 2025, 16, 2589. 40091058 10.1038/s41467-025-57800-y PMC11911444

[36] Y. Gu , W. Wu , C. Zhang , X. Li , X. Guo , Y. Wang , Y. Yuan , B. Jiang , Y. Jin , Adv. Funct. Mater. 2025, 35, 2417206.

[37] X. Duan , W. Cao , X. He , M. Wang , R. Cong , Z. Zhang , C. Ning , C. Wang , S. Zhao , Z. Li , W. Gao , Chem. Eng. J. 2023, 476, 146536.

[38] H. Li , J.‐T. Sun , C. Wang , S. Liu , D. Yuan , X. Zhou , J. Tan , L. Stubbs , C. He , ACS Sustainable Chem. Eng. 2017, 5, 7942.

[39] F. Zhang , A. H. Barber , Macromol. Mater. Eng. 2017, 302, 1700084.

[40] F. Yang , J. Zhao , W. J. Koshut , J. Watt , J. C. Riboh , K. Gall , B. J. Wiley , Adv. Funct. Mater. 2020, 30, 2003451.

[41] X. Hu , M. Vatankhah‐Varnoosfaderani , J. Zhou , Q. Li , S. S. Sheiko , Adv. Mater. 2015, 27, 6899. 26436409 10.1002/adma.201503724

[42] Q. He , Y. Huang , S. Wang , Adv. Funct. Mater. 2018, 28, 1705069.

[43] A. K. Means , M. A. Grunlan , ACS Macro Lett. 2019, 8, 705. 33912358 10.1021/acsmacrolett.9b00276 PMC8077972

[44] F. Lin , Z. Wang , J. Chen , B. Lu , L. Tang , X. Chen , C. Lin , B. Huang , H. Zeng , Y. Chen , J. Mater. Chem. B 2020, 8, 4002. 32227057 10.1039/d0tb00424c

[45] L. Zhang , J. Zhao , J. Zhu , C. He , H. Wang , Soft Matter 2012, 8, 10439.

[46] Y.a‐N. Chen , L. Peng , T. Liu , Y. Wang , S. Shi , H. Wang , ACS Appl. Mater. Interfaces. 2016, 8, 27199. 27648478 10.1021/acsami.6b08374

[47] Q. Liu , X. Dong , H. Qi , H. Zhang , T. Li , Y. Zhao , G. Li , W. Zhai , Nat. Commun. 2024, 15, 3237. 38622154 10.1038/s41467-024-47597-7 PMC11018840

[48] D. Ji , Z. Zhang , J. Sun , W. Cao , Z. Wang , X. Wang , T. Cao , J. Han , J. Zhu , ACS Appl. Mater. Interfaces. 2024, 16, 25304. 38654450 10.1021/acsami.4c02354

[49] Q. Zhou , J. Lyu , G. Wang , M. Robertson , Z. Qiang , B. Sun , C. Ye , M. Zhu , Adv. Funct. Mater. 2021, 31, 2104536.

[50] J. Li , Z. Suo , J. J. Vlassak , J. Mater. Chem. B 2014, 2, 6708. 32261867 10.1039/c4tb01194e

[51] J. Lyu , X. Wang , L. Liu , Y. Kim , E. K. Tanyi , H. Chi , W. Feng , L. Xu , T. Li , M. A. Noginov , C. Uher , M. D. Hammig , N. A. Kotov , Adv. Funct. Mater. 2016, 26, 8435.

[52] J.‐Y. Sun , X. Zhao , W. R. K. Illeperuma , O. Chaudhuri , K. H. Oh , D. J. Mooney , J. J. Vlassak , Z. Suo , Nature 2012, 489, 133. 22955625 10.1038/nature11409 PMC3642868

[53] X. Zhang , W. Liu , J. Cai , J. Huang , X. Qiu , J. Mater. Chem. A 2019, 7, 26917.

[54] F. Wang , Z. Li , J. Guo , L. Liu , H. Fu , J. Yao , I. Krucinska , Z. Draczynski , ACS Appl. Polym. Mater. 2022, 4, 618.

