# Supplementary material for: Ultra‐Robust Conductive Hydrogels Enabled by a Gradient Bond‐Breaking Pseudo‐Drying Strategy
Source: Adv Sci (Weinh). 2025 Sep 29;12(45):e12144. doi: 10.1002/advs.202512144 (PMC12677661; doi:10.1002/advs.202512144)
Supplement: Supplementary file 1 — Supporting Information [file ADVS-12-e12144-s001.docx]

Supporting Information

**Ultra-Robust Conductive Hydrogels Enabled by a Gradient Bond-Breaking Pseudo-Drying Strategy**

*Dongchao Ji, Hongyang Han, Jiajun Li, Xiaoman Fei, Xiaolei Wang, Zhuochao Wang, Tao Song, Lei Yang, Zhibo Zhang,* Wenxin Cao,* Jiecai Han, Jiaqi Zhu**

Dongchao Ji, Jiajun Li, Xiaoman Fei, Xiaolei Wang, Zhuochao Wang, Fei, Lei Yang, Zhibo Zhang, Wenxin Cao, Jiecai Han, Jiaqi Zhu

National Key Laboratory of Science and Technology on Advanced Composites in Special Environments

Harbin Institute of Technology

Harbin 150001, P. R. China

E-mail: zhujq@hit.edu.cn; caowenxin@hit.edu.cn

Zhibo Zhang, Wenxin Cao
Zhengzhou Research Institute

Harbin Institute of Technology

Zhengzhou 450000, P. R. China

E-mail: zbzhang@hit.edu.cn

Hongyang Han, Tao Song

School of Stomatology

Harbin Medical University

Harbin 150081, P. R. China

Figure S1-S1 showing TEM of aramid nanofibers (ANFs); SEM image of protonated ANFs; Tensile stress–strain curves of the effect of PVA concentration on the mechanical properties of H-P hydrogels; Tensile stress–strain curves of S-P15A3 hydrogel in the parallel (‖) and perpendicular (⊥) directions relative to the alignment direction; Fracture toughness of H-P; 65% compressive stress−strain curves of H-P and P-PA hydrogels; 10 compression cycles of P-PA hydrogel at 40% compressive strain; G’, G”, and tan δ of H-P gels from strain amplitude sweep (10−33%) at a fixed angular frequency (10 rad/s); G’, G”, and tan δ of S-PA gels from strain amplitude sweep (10−33%) at a fixed angular frequency (10 rad/s); Mechanical properties of the P-PA hydrogel with decreasing temperature; Changes in water content of P-PA and H-P hydrogels exposed to air with time.

Table S1: Mechanical comparison between the present work and different hydrogels.


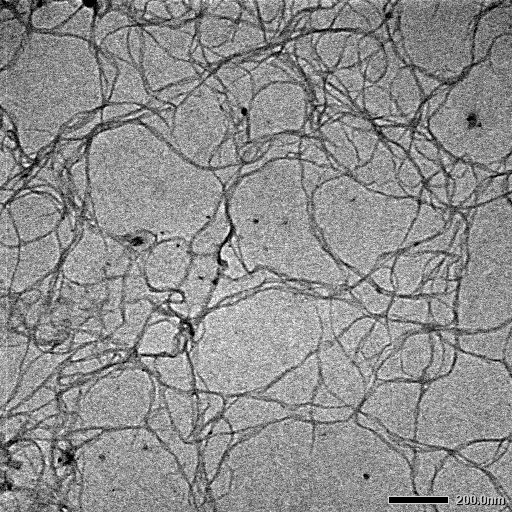


**Figure S1. TEM of aramid nanofibers (ANFs).**


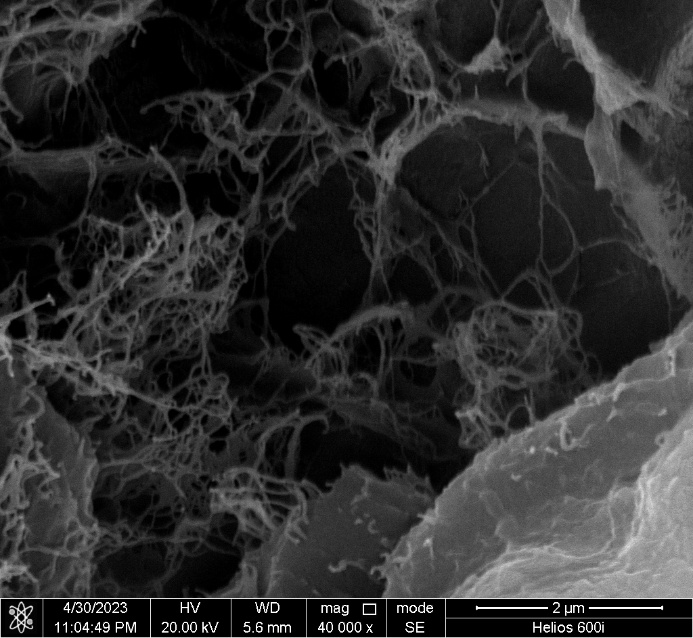


**Figure S2. SEM image of protonated ANFs.**


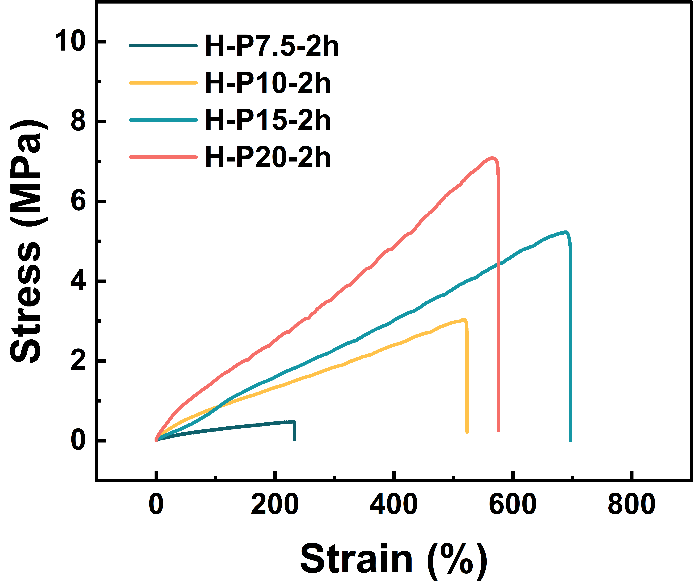


**Figure S3. Tensile stress–strain curves of the effect of PVA concentration on the mechanical properties of H-P hydrogels.**


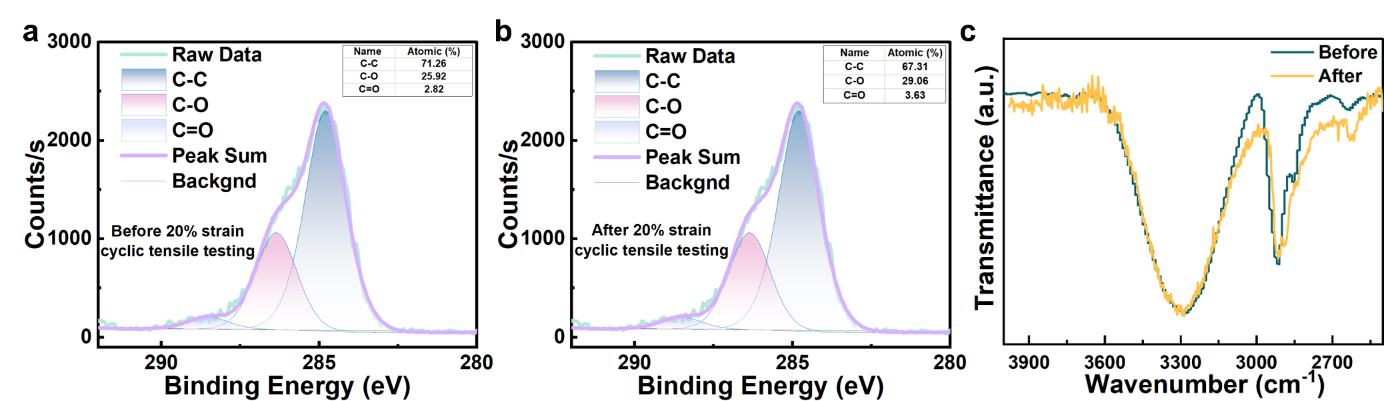


**Figure S4. Verification of gradient bonding breakage. (a) XPS of samples before 20% low strain cycling. (b) XPS of samples after 20% low strain cycling. (c) FT-IR for corresponding samples.**


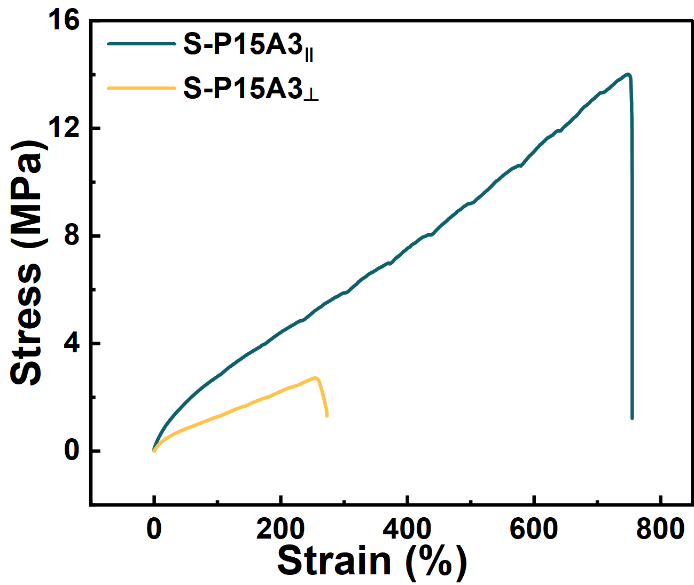


**Figure S5. Tensile stress–strain curves of S-P15A3 hydrogel in the parallel (‖) and perpendicular (⊥) directions relative to the alignment direction.**


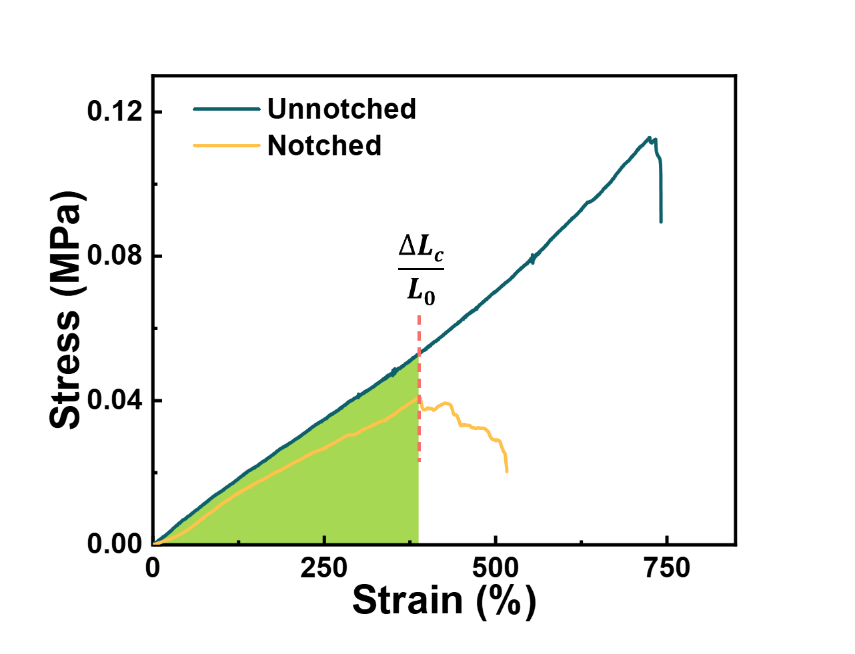


**Figure S6. Fracture toughness of H-P.**


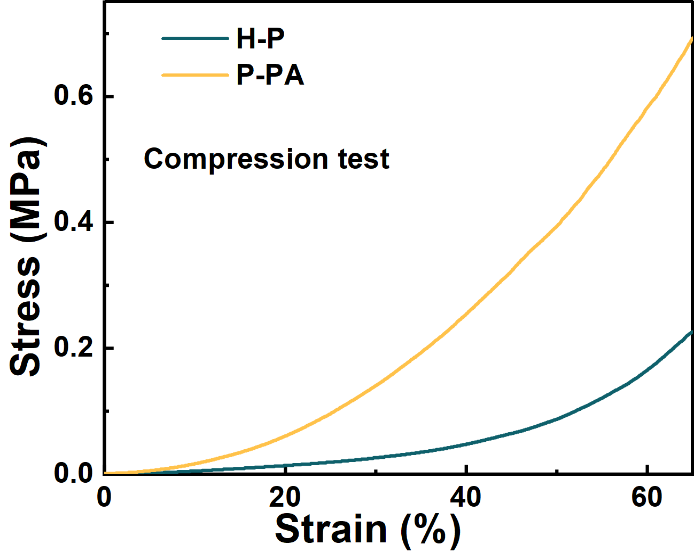


**Figure S7. 65% compressive stress−strain curves of H-P and P-PA hydrogels.**


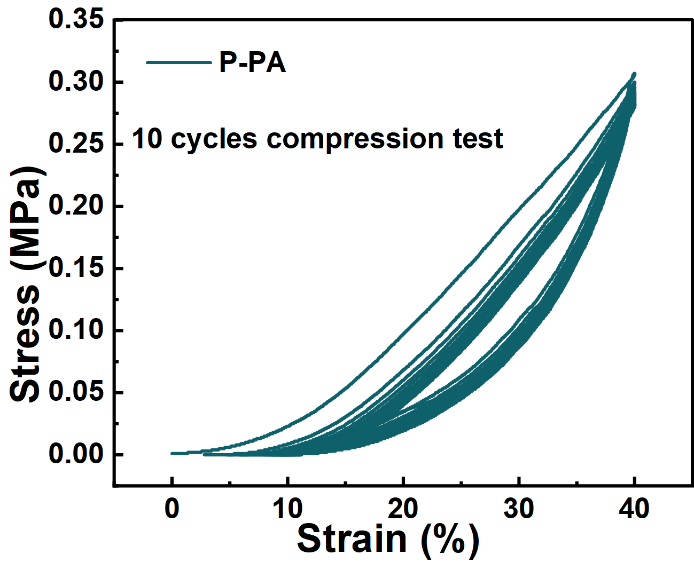


**Figure S8. 10 compression cycles of P-PA hydrogel at 40% compressive strain.**


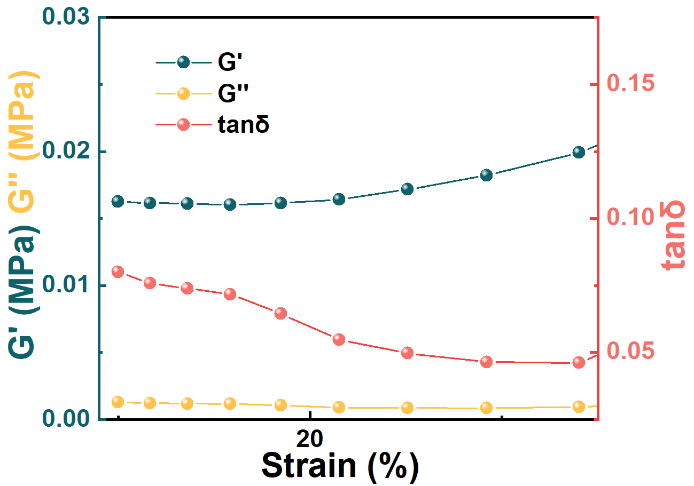


**Figure S9. G’, G”, and tan δ of H-P gels from strain amplitude sweep (10−33%) at a fixed angular frequency (10 rad/s).**

**Figure S10. G’, G”, and tan δ of S-PA gels from strain amplitude sweep (10−33%) at a fixed angular frequency (10 rad/s).**


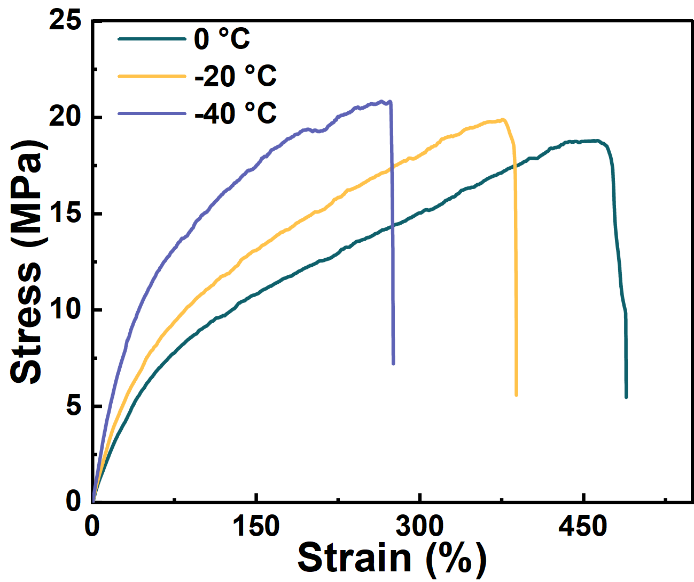


**Figure S11. Mechanical properties of the P-PA hydrogel with decreasing temperature.**


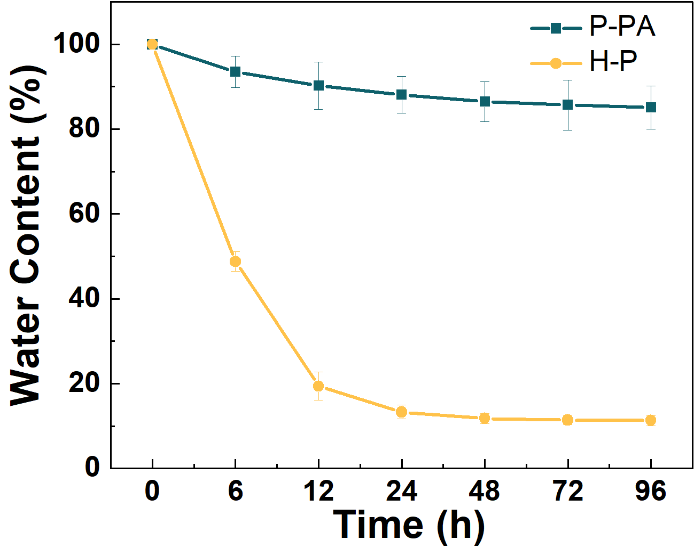


**Figure S12. Changes in water content of P-PA and H-P hydrogels exposed to air with time.**

**Table S1. Mechanical comparison between the present work and different hydrogels.**

| **Hydrogel type** | **Reference** | **Tensile strength**  **(MPa)** | **Mdulus**  **(MPa)** | **Toughness**  **(MJ/m^3^)** | **Fracture energy**  **(kJ/m^2^)** |
| --- | --- | --- | --- | --- | --- |
| **PVA exogel** | ^[^[^1^](#_ENREF_1)^]^ | 3 | 0.46 | 13.5 | 3.72 |
|  |  | 1 | 0.17 | 4.1 | 1.5 |
|  |  | 2 | 0.17 | 3.38 | 1.6 |
|  |  | 0.5 | 0.4 | 1 | 0.8 |
| **Cartilage** | ^[^[^2^](#_ENREF_2)^]^ | 10 | 1 | 0.15 | 1 |
| **pva/anf** | ^[^[^3^](#_ENREF_3)^]^ | 5 | 9.1 | 9 | 9.2 |
|  |  | 1.4–5.0 | 1.9–9.1 | 0.35-10 | 2.3-9.2 |
| **S-PVA** | ^[^[^4^](#_ENREF_4)^]^ | 21 | 6.5 | 50 | 4.5 |
|  |  | 9 | 1.7 | 12 | 1.3 |
|  |  | 13 | 4 | 30 | 4 |
| **PVA/Ta/CNC** | ^[^[^5^](#_ENREF_5)^]^ | 3 | 0.7 | 8 |  |
|  |  | 1.6 | 0.36 | 2 |  |
|  |  | 8.6 | 2 | 50 |  |
|  |  | 3.9 | 0.8 | 17 |  |
| **pva** | ^[^[^6^](#_ENREF_6)^]^ | 0.3 | 0.03 |  | 0.00017 |
|  |  | 1.2 | 0.1 |  | 0.0004 |
| **pva-ta** | ^[^[^7^](#_ENREF_7)^]^ | 0.5 | 0.11 |  |  |
|  |  | 1 | 0.2 |  |  |
|  |  | 2 | 0.3 |  |  |
|  |  | 3 | 0.35 |  |  |
| **Trained PVA** | ^[^[^8^](#_ENREF_8)^]^ | 1 | 0.14 |  | 0.0006 |
|  |  | 5 | 0.26 |  | 0.0016 |
| **3D printable PVA** | ^[^[^9^](#_ENREF_9)^]^ | 7.81 | 9.4 | 18.1 | 20.3 |
|  |  | 6.8 | 4.93 | 14.5 | 31.1 |
| **PVA/PVP/ANFs** | ^[^[^10^](#_ENREF_10)^]^ | 5.26 | 0.84 | 17.69 | 1.47 |
|  |  | 9.76 | 2.17 | 31.84 | 4.12 |
| **PVA/ANFs/AgNWs** | ^[^[^11^](#_ENREF_11)^]^ | 0.7 | 0.4 | 1 | 0.45 |
|  |  | 3.35 | 10.8 | 2.26 | 5.7 |
|  |  | 3.9 | 12.4 | 1.18 | 4.8 |
|  |  | 5.6 | 15.4 | 0.94 | 4.7 |
| **Dry-anneal PVA/PAAm** | ^[^[^12^](#_ENREF_12)^]^ | 2.5 | 5 | 4.53 | 14 |
|  |  | 4.7 | 12 | 9.17 | 19 |
|  |  | 1.6 | 3 | 3.7 | 8 |
|  |  | 1.2 | 2.5 | 1.95 | 5 |
|  |  | 0.3 | 0.25 | 0.43 | 0.8 |
| **P-PA** | This work | 17.785 | 12.4 | 73.66 | 268.8 |
|  |  | 14.0162 | 4.63 | 55.35 |  |
|  |  | 15.67875 | 9.28 | 69.95 |  |
|  |  | 11.3525 | 17.64 | 4.98 |  |

**References**

1 L. Xu, S. Gao, Q. Guo, et al., "A Solvent-Exchange Strategy to Regulate Noncovalent Interactions for Strong and Antiswelling Hydrogels," *Adv. Mater.* vol. 32, no. 52 (2020): 2004579. <https://doi.org/10.1002/adma.202004579>

2 A. K. Means, and M. A. Grunlan, "Modern Strategies To Achieve Tissue-Mimetic, Mechanically Robust Hydrogels," *ACS Macro Lett.* vol. 8, no. 6 (2019): 705-713. 10.1021/acsmacrolett.9b00276

3 L. Xu, X. Zhao, C. Xu, and N. A. Kotov, "Water-Rich Biomimetic Composites with Abiotic Self-Organizing Nanofiber Network," *Adv. Mater.* vol. 30, no. 1 (2018): 1703343. <https://doi.org/10.1002/adma.201703343>

4 D. Liu, Y. Cao, P. Jiang, et al., "Tough, Transparent, and Slippery PVA Hydrogel Led by Syneresis," *Small* vol. 19, no. 14 (2023): 2206819. <https://doi.org/10.1002/smll.202206819>

5 F. Lin, Z. Wang, J. Chen, et al., "A bioinspired hydrogen bond crosslink strategy toward toughening ultrastrong and multifunctional nanocomposite hydrogels," *J. Mater. Chem. B* vol. 8, no. 18 (2020): 4002-4015. 10.1039/D0TB00424C

6 L. Zhang, J. Zhao, J. Zhu, et al., "Anisotropic tough poly(vinyl alcohol) hydrogels," *Soft Matter* vol. 8, no. 40 (2012): 10439-10447. 10.1039/C2SM26102B

7 Y.-N. Chen, L. Peng, T. Liu, et al., "Poly(vinyl alcohol)–Tannic Acid Hydrogels with Excellent Mechanical Properties and Shape Memory Behaviors," *ACS Appl. Mater. Interfaces* vol. 8, no. 40 (2016): 27199-27206. 10.1021/acsami.6b08374

8 S. Lin, J. Liu, X. Liu, and X. Zhao, "Muscle-like fatigue-resistant hydrogels by mechanical training," *Proceedings of the National Academy of Sciences* vol. 116, no. 21 (2019): 10244-10249. 10.1073/pnas.1903019116

9 Q. Liu, X. Dong, H. Qi, et al., "3D printable strong and tough composite organo-hydrogels inspired by natural hierarchical composite design principles," *Nat. Commun.* vol. 15, no. 1 (2024): 3237. 10.1038/s41467-024-47597-7

10 D. Ji, Z. Zhang, J. Sun, et al., "Strong, Tough, and Biocompatible Poly(vinyl alcohol)–Poly(vinylpyrrolidone) Multiscale Network Hydrogels Reinforced by Aramid Nanofibers," *ACS Appl. Mater. Interfaces* vol. 16, no. 19 (2024): 25304-25316. 10.1021/acsami.4c02354

11 Q. Zhou, J. Lyu, G. Wang, et al., "Mechanically Strong and Multifunctional Hybrid Hydrogels with Ultrahigh Electrical Conductivity," *Adv. Funct. Mater.* vol. 31, no. 40 (2021): 2104536. <https://doi.org/10.1002/adfm.202104536>

12 J. Li, Z. Suo, and J. J. Vlassak, "Stiff, strong, and tough hydrogels with good chemical stability," *J. Mater. Chem. B* vol. 2, no. 39 (2014): 6708-6713. 10.1039/C4TB01194E
